# MLVA Based Classification of *Mycobacterium tuberculosis* Complex Lineages for a Robust Phylogeographic Snapshot of Its Worldwide Molecular Diversity

**DOI:** 10.1371/journal.pone.0041991

**Published:** 2012-09-11

**Authors:** Véronique Hill, Thierry Zozio, Syndia Sadikalay, Sofia Viegas, Elisabeth Streit, Gunilla Kallenius, Nalin Rastogi

**Affiliations:** 1 WHO Supranational TB Reference Laboratory, TB and Mycobacteria Unit, Institut Pasteur de la Guadeloupe, Abymes, France; 2 National Institute of Health, Ministry of Health, Maputo, Mozambique; 3 Department of Clinical Science and Education, Södersjukhuset, Karolinska Institutet, Stockholm, Sweden; University of Padova, Italy

## Abstract

Multiple-locus variable-number tandem repeat analysis (MLVA) is useful to establish transmission routes and sources of infections for various microorganisms including *Mycobacterium tuberculosis* complex (MTC). The recently released SITVITWEB database contains 12-loci Mycobacterial Interspersed Repetitive Units – Variable Number of Tandem DNA Repeats (MIRU-VNTR) profiles and spoligotype patterns for thousands of MTC strains; it uses MIRU International Types (MIT) and Spoligotype International Types (SIT) to designate clustered patterns worldwide. Considering existing doubts on the ability of spoligotyping alone to reveal exact phylogenetic relationships between MTC strains, we developed a MLVA based classification for MTC genotypic lineages. We studied 6 different subsets of MTC isolates encompassing 7793 strains worldwide. Minimum spanning trees (MST) were constructed to identify major lineages, and the most common representative located as a central node was taken as the prototype defining different phylogenetic groups. A total of 7 major lineages with their respective prototypes were identified: Indo-Oceanic/MIT57, East Asian and African Indian/MIT17, Euro American/MIT116, West African-I/MIT934, West African-II/MIT664, *M. bovis*/MIT49, *M.canettii*/MIT60. Further MST subdivision identified an additional 34 sublineage MIT prototypes. The phylogenetic relationships among the 37 newly defined MIRU-VNTR lineages were inferred using a classification algorithm based on a bayesian approach. This information was used to construct an updated phylogenetic and phylogeographic snapshot of worldwide MTC diversity studied both at the regional, sub-regional, and country level according to the United Nations specifications. We also looked for IS*6110* insertional events that are known to modify the results of the spoligotyping in specific circumstances, and showed that a fair portion of convergence leading to the currently observed bias in phylogenetic classification of strains may be traced back to the presence of IS*6110.* These results shed new light on the evolutionary history of the pathogen in relation to the history of peopling and human migration.

## Introduction

The most commonly used genotyping methods to characterize the circulating *Mycobacterium tuberculosis* complex (MTC) clones in various populations are PCR-based spoligotyping [1] and Mycobacterial Interspersed Repetitive Units – Variable Number of Tandem DNA Repeats (MIRU-VNTR) minisatellites [2], [3], [4], that constitute the backbone of one of the biggest publicly available MTC genotyping database SITVITWEB released in 2012 [5]. The spoligotyping-based classification of MTC clinical isolates was used for a first description of 62 MTC genotypic lineages/sublineages in the SpolDB4 database [6]. It was based on the assumption of a unidirectional evolution of strains by loss of spacers in the direct repeat (DR) locus. Despite its flaws [7], spoligotyping based classification fits quite well with an overall evolutionary picture of MTC using the *katG-gyrA* polymorphism [8] and the presence of a specific deletion region (TbD1; [9]). The former method allowed subdivision of MTC isolates into 3 principal genetic groups (PGG; PGG1 strains being evolutionarily older than PGG2/3 isolates), while the later study underlined that ancestral MTC isolates are TbD1^+^ as opposed to TbD1^–^ modern isolates.

As reviewed recently [5], [7], the various spoligotyping-defined lineages fit well in 3 large phylogenetical groups: ancestral TbD1^+^/PGG1 group (East African Indian, EAI), modern TbD1^–^/PGG1 group (Beijing and Central Asian or CAS), and evolutionary recent TbD1–/PGG2/3 group (Haarlem, X, S, T, and Latin American and Mediterranean or LAM). However, proper epidemiologic and phylogenetic inferences are not always an easy task due to a lack of understanding of the mechanisms behind the mutations leading to the polymorphism of these genomic targets. Recent studies have shown that phylogenetically unrelated MTC strains could be found with the same spoligotype pattern as a result of independent mutational events [10], an observation that corroborates the fact that spoligotyping is prone to homoplasy to a higher extent than the MIRU-VNTRs [11]. Furthermore, spoligotyping has little discriminative power for families associated with the absence of large blocks of spacers, e.g., the Beijing lineage defined by its prototype – spoligotyping international type 1 (SIT1) in the SpolDB4 database.

The usefulness of minisatellite-based lineage classification of MTC isolates was attempted by Allix-Béguec et al. [12], who described a web-based server with detailed information on a well-characterized set of 186 reference isolates; each strain being described for its geographical origin, corresponding genetic lineage, IS*6110*-RFLP, 24-locus MIRU-VNTR, spoligotyping, Single Nucleotide Polymorphism (SNPs), and Large Sequence Polymorphism (LSP) profiles (http://www.MIRU-VNTRplus.org). The authors described and tested an algorithm based on best-match analysis followed by tree-based analysis on MIRU-VNTR data (combined or not with spoligotyping data) to describe distribution of isolates with minisatellite data among the various spoligotype families. However, the authors did not interpret their data to describe minisatellite-based lineages, since conclusions were essentially drawn based on spoligotype-based classification.

Considering existing doubts on the ability of spoligotyping alone to reveal exact phylogenetic relationships between MTC strains [11], [13], particularly the classification of evolutionary recent TbD1–/PGG2/3 group [14]; we decided to study 6 different subsets of MTC isolates encompassing 7793 strains (see subsection 2 of “Materials and Methods” for information on the origin of the strains used). The purpose of this paper is to: (i) classify these strains based on 12 locus MIRU-VNTR typing data; (ii) to draw the evolutionary history of various MTC members (species, subspecies, groups) leading to the diversity of newly described phylogenetic lineages/groups; (iii) to see how the geographical distribution of these lineages reinforces the history of human settlement in the world, and finally, (iv) to evaluate the MLVA based classification of MTC genotypic lineages as a means to provide with an accurate and robust phylogeographic interpretation of its worldwide diversity.

**Table 1:** Description of the 7 major lineages and 41 sublineages based on 12-loci MIRU-VNTRs.

MIRU-VNTR lineages/Central node MIT	Sublineages/Central Node MIT	12-loci MIRU-VNTR patterns of Central Node MITs	Corresponding LSP-based lineages	Corresponding Spoligotype lineages	Spoligotype rule (absence of spacers)	SIT Number
Indo-Oceanic/MIT57	Indo-Oceanic-57	254326223533	Indo-Oceanic	East African- Indian (EAI)	29–32,34	236
	Indo-Oceanic-56	254326223432				
	Indo-Oceanic-59	264225223533				
	Indo-Oceanic-64	254326223513				
	Indo-Oceanic-69	254326223434				
East Asian and African Indian/MIT17	East Asian-17	223325173533	East Asian	Beijing	1–34	1
	East Asian-16	223325153533				
	East Asian-83	223325163533				
	East Asian-86	223325173433				
	East Asian-93	223425173533				
	East Asian-99	223325173543				
	East Asian-101	223325173523				
	East-African Indian-68	225425173533	East-African Indian	Central-Asian (CAS)	4–7,23–34	26
	East-African Indian-261	227425113434				
Euro American/MIT116	Euro American-116	223325153323	Euro American	Haarlem, Latin American and Mediterranean (LAM), X, T, S	33–36	53
	Euro American-7	222325153323				
	Euro American-8	223125153324				
	Euro American-12	223315153323				
	Euro American-15	223325153322				
	Euro American-25	224226153321				
	Euro American-33	224325153323				
	Euro American-34	224325153324				
	Euro American-40	225125113322				
	Euro American-42	225313153323				
	Euro American-43	225323153323				
	Euro American-45	225325153323				
	Euro American-46	225325153324				
	Euro American-112	223325143324				
	Euro American-121	223325143323				
	Euro American-125	223325153324				
	Euro American-128	223226153321				
	Euro American-163	224126152321				
	Euro American-190	124326153220				
	Euro American-212	233325153324				
	Euro American-213	224326153323				
	Euro American-224	223326153321				
	Euro American-246	124326153324				
West African I/MIT934	West African I - 934	224424244221	West African lineage I	AFRI2, AFRI3	8-12, 37–39	438
West African II/MIT664	West African II – 664	236424253522	West African lineage II	AFRI1	7–9, 39	181
			–	BOV_4-CAPRAE	1,3, 16, 28, 39–43	647
*M. bovis/*MIT49	*M. bovis – 49*	232324253322	–	*M. bovis*	3, 16, 39–43	3090
*M. canettii/*MIT60	*M. canettii – 60*	323212632428	–	*M. canettii*	30.36	592

These lineages/sublineages were identified from a MST tree constructed with 7009 strains taken from the SITVIT2 proprietary database of Institut Pasteur de la Guadeloupe. The corresponding LSP-based lineages [23] and Spoligotype-based lineages [6] are shown for comparison.

## Materials and Methods

### 1. Molecular methods

This investigation made use of available genotyping data of *Mycobacterium tuberculosis* complex (MTC) clinical isolates using standard spoligotyping and MIRU-VNTR typing techniques [1], [3], [4], and the reader is referred to subsection 2 below for information on the origin of the strains and published data used. In selected cases, we further checked for blocks of deleted spacers in the standard 43-spacer spoligotyping format by extended spoligotyping using methodology described earlier [15], [16]. For this purpose, 2 additional membranes were used to reveal the presence or absence of spacers 1 to 86 in the genomic order established on the *M. tuberculosis* H37Rv reference strain [17].

**Figure 1:**
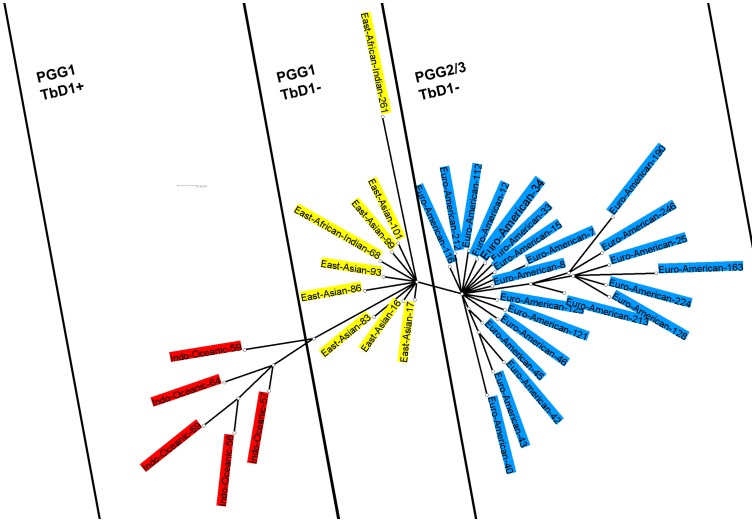
Phylogenetic tree constructed with MrBayes3 software ( http://mrbayes.csit.fsu.edu/ **).** The tree is done with the 37 MIRU-VNTR prototypes of *M. tuberculosis* sensu stricto.

We also looked for IS*6110* insertional events that are known to modify the results of the spoligotyping in specific circumstances; briefly, we used pairs of primers (biotinylated)DRa-IS3, (biot)DRb-IS6, (biot)DRa-IS6 and (biot)DRb-IS3, to highlight the presence of IS*6110* [18], [19]. Additionally, an IS*6110* Adjacent Deletion Typing (IS*6110*AD-typing) was developed in-house to investigate the role of IS*6110* insertional event(s) causing deletions in the MTC genome elsewhere than the DR locus. The phenomenon of “adjacent deletion” in which a contiguous chromosomal segment adjacent to the transposon is deleted while the element responsible remains intact, was initially described by Roberts et al. [20]. For this purpose, we identified 16 copies of IS*6110* in *M. tuberculosis* H37Rv genome (reference sequence NC_000962, NCBI genome database). Note that the 11^th^ copy located in the DR locus was not retained due to the well-known variability of this locus in relation to insertional events (see above), and the fact that it constitutes a hotspot for IS*6110* insertional preferential locus (*ipl*; [21]). Consequently, IS*6110*AD-typing targeted regions adjacent to 15 IS*6110* copies leading to the final amplification of 28 genomic sequences (2 copies of IS*6110* were contiguous, with no amplification between them). Please refer to Tables S1 and S2 for the description of primers used and for IS*6110*AD-typing and experimental conditions, respectively.

**Table 2:** Comparison of the new MIRU-VNTR based lineages with the Brudey's classification.

MIRU-VNTR lineages/Central node MIT	Sublineages/Central Node MIT	Corresponding Spoligotype lineages (proportion)	Corresponding Spoligotype sublineages (proportion)
Indo-Oceanic/MIT57	Indo-Oceanic-57	98.53% EAI	42.65% EAI5
	Indo-Oceanic-56	97.14% EAI	77.14% EAI2-Manila
	Indo-Oceanic-59	100% EAI	57.5% EAI5
	Indo-Oceanic-64	97.67% EAI	68.60% EAI1-SOM
	Indo-Oceanic-69	97.37% EAI	72.97% EAI3-IND
East Asian, African Indian/MIT17	East Asian-17	97.64% Beijing	
	East Asian-16	97.86% Beijing	
	East Asian-83	99.01% Beijing	
	East Asian-86	96.72% Beijing	
	East Asian-93	85.71% Beijing	
	East Asian-99	100% Beijing	
	East Asian-101	63.33% Beijing	
	East-African Indian-68	94.12% CAS	68.07% CAS1-Delhi
	East-African Indian-261	87.10% CAS	74.19% CAS1-Kili

This table concerns only two MIRU12-based lineages: Indo-Oceanic and East Asian and African Indian.

### 2. Genotyping information

This investigation made use of available genotyping data or in-house typing of six different subsets of *Mycobacterium tuberculosis* complex (MTC) clinical isolates encompassing 7793 strains of diverse geographical origin as follows:

**Figure 2:**
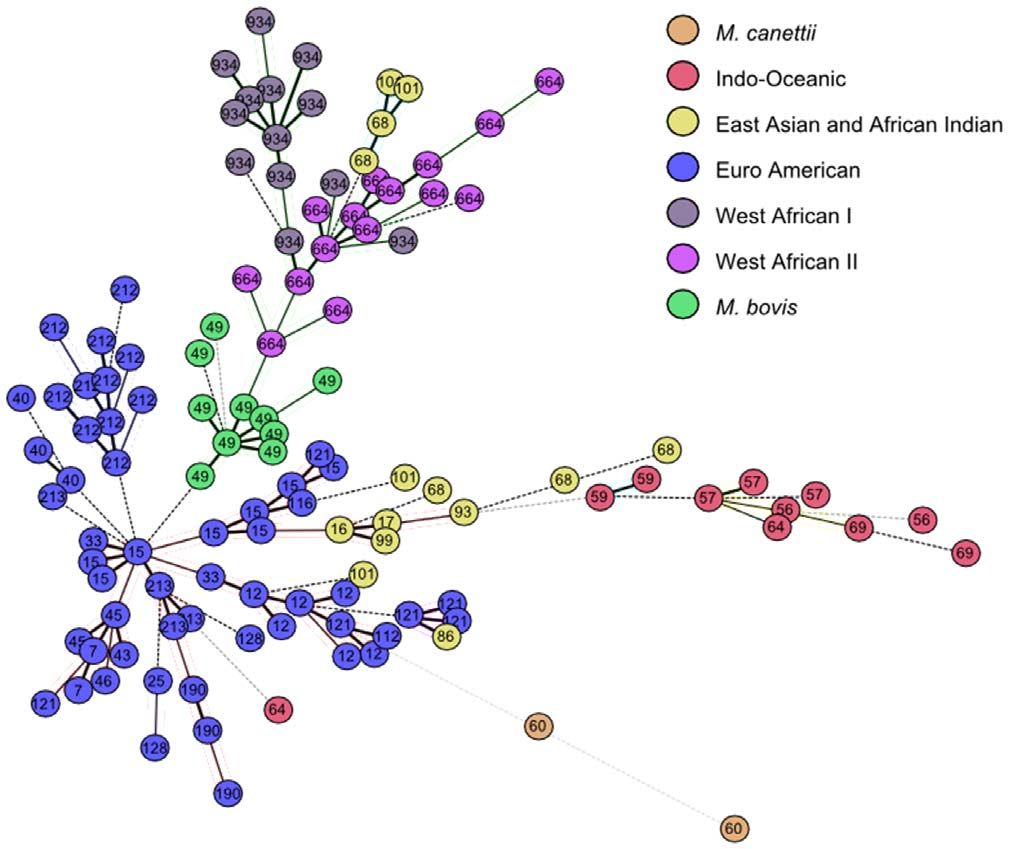
MST tree done with 12 MIRU-VNTR loci of 176 strains from MIRU-VNTR*plus* **database** (http://www.miru-vntrplus.org/MIRU/index.faces).

- Spoligotyping and 12-loci MIRU-VNTR data on 7009 strains from the SITVIT2 proprietary database of Institut Pasteur de la Guadeloupe (n = 5990 strains genotyped by various investigators, list available through http://www.pasteur-guadeloupe.fr:8081/SITVIT_ONLINE; n = 1019 strains genotyped at Institut Pasteur de la Guadeloupe as follows: Guadeloupe n = 203; Martinique n = 88; French Guiana n = 364; Dominican Republic n = 88; Colombia n = 134; and Turkey n = 142). This dataset was used to establish the 12-locus MIRU-VNTR rules, followed by their validation in other datasets described below.
- Genotypic data on 176 MTC isolates from the MIRU-VNTRplus database (http://www.miru-vntrplus.org/MIRU/index.faces). The aim of this selection (Table S3) was to compare the MLVA based classification of MTC strains developed during this study versus previous labeling using SpolDB4 [6] and LSP-based classification [22], [23]. Note that data on *M. microti* and *M. pinnipedii* isolates was set aside since they were almost inexistent in the subset 1 (no *M. microti* strains, and only 1 *M. pinnipedii* among the 7009 strains initially used to establish the 12-locus MIRU-VNTR rules).
- The MIRU-VNTR rules were further evaluated on a subset of LAM strains to describe the novel RD^rio^ lineage [24] (Table S4; n = 190). This group was subdivided in 2 subgroups: 100 strains with RD^rio^ deletion and 90 wild-type strains.
- To test a hypothesis about an Asia-to-Africa back migration theory based on the study of Y-chromosome haplogroups at Neolithic times [25], we also used published data on 154 MTC strains from the north west of Iran [26].
- To compensate the lack of MIRU-VNTR data on MTC isolates from East-Africa in all published genotyping databases, we decided to type strains from Mozambique. For this purpose, 100 MTC clinical isolates were blindly sampled starting from an initial set of 445 clinical isolates studied recently using spoligotyping in Mozambique [27]. These isolates were typed using 24-loci MIRU-VNTRs, extended spoligotyping, the detection of IS*6110* insertions in the DR locus, and IS*6110*AD-typing as described above under the subsection 1.
- Lastly, the principle of lineage identification developed was initially validated on a set of 164 strains typed by spoligotyping and 12-loci MIRU-VNTRs from Kerala (unpublished data).

**Table 3:** Some cases of discrepancies in classification between the newly defined lineages based on 12-loci MIRU-VNTRs and the Brudey's classification scheme in SpolDB4 (see footnote for extended explanation).

Case	12-loci MIRU-VNTR pattern	MIT b	Sub case N°	Spoligotype43 pattern	Brudey's classification	MIRU-VNTR based classification
A	254326223432	56	1	▪▪□▪▪▪▪□□□□□□□□□□□□□□□□□□▪▪▪□□□□▪□▪▪▪▪▪▪▪▪▪	EAI2-Nonthaburi	Indo-Oceanic-56
	254326223432	56	2	▪□□▪▪▪▪□□□□□□□□□□□□□□□□□□▪▪▪□□□□▪□▪▪▪▪▪▪▪▪▪	EAI5	Indo-Oceanic-56
B	254326223514	78	1	▪▪▪▪▪▪▪▪▪▪▪▪▪▪▪▪▪▪▪▪▪▪▪▪▪▪▪▪□□□□▪□▪▪▪▪▪□▪▪▪	EAI1-SOM	Indo-Oceanic-64
	234326223513	629	2	▪▪▪▪▪▪▪▪▪▪▪▪▪▪▪▪▪▪▪▪▪▪▪▪▪▪▪▪□□□□▪□▪▪▪▪▪□□▪▪	EAI5	Indo-Oceanic-64
C	226425153533	271	1	▪▪▪□□□□▪▪▪▪▪▪▪▪▪▪▪▪▪▪▪□□□□□□□□□□□□▪▪□□▪▪▪▪▪	CAS1-Delhi	East-African Indian-68
	226425153633	413	2	▪▪▪□□□□▪▪▪▪▪▪▪▪▪▪▪▪▪▪▪□□□□□□□□□□▪□▪▪□□▪▪▪▪▪	EAI5	East-African Indian-68
D	224126152321	163	1	▪▪▪▪▪▪▪▪▪▪▪▪▪▪▪▪▪▪▪▪□□□□▪▪□□□□▪▪□□□□▪▪▪▪▪▪▪	LAM11-ZWE	Euro American-163
	224126152321	163	2	▪▪▪▪▪▪▪▪▪▪▪▪▪▪▪▪▪▪▪▪□□▪□▪▪□□□□▪▪□□□□▪▪▪▪▪▪▪	T1	Euro American-163
E	124325153225	1	1	▪▪▪▪▪▪▪▪▪▪▪▪▪▪□□□□□□□□□□▪▪▪▪▪▪▪▪□□□□▪▪▪▪▪▪▪	T5-RUS1	Euro American-190
	124326153224	140	2	▪▪▪▪▪▪▪▪▪▪▪▪▪▪□□□□□□□□□▪▪▪▪▪▪▪▪▪□□□□▪▪▪▪▪▪▪	T-Tuscany	Euro American-190
F^a^	215125113322	310	1	▪▪▪▪▪▪▪▪▪▪▪▪▪▪▪▪▪▪▪□□□□□▪□□▪▪▪▪▪□□□□▪▪▪▪▪▪▪	LAM7-TUR	Euro American-40
	226125113322	430	2	▪▪▪▪▪▪▪▪▪□□□□□□□□□□▪▪▪▪▪▪▪▪▪▪▪▪▪□□□□▪▪▪▪▪▪▪	T3-ETH	Euro American-40
G	227425113434	261	1	▪▪▪□□□□▪▪□▪▪▪▪▪▪▪▪▪□□□□□□□□□□□□□□□□▪▪▪▪▪▪▪▪	CAS1-Kili	East-African Indian-261
	227225113224	200	2	▪▪▪▪▪▪▪□□▪▪▪▪▪▪▪▪▪▪□□□□□□□□□□□□▪□□□□▪▪▪▪▪▪▪	H3	East-African Indian-261
H	254326223334	577	1	□▪▪□□▪▪▪▪▪▪▪▪▪▪▪▪▪▪▪▪▪▪▪▪▪▪▪□□□□▪□▪▪▪▪▪□▪▪▪	EAI1-SOM	Indo-Oceanic-69
	254326223424	543	2	▪□□▪▪▪▪▪▪▪▪▪▪▪▪▪▪▪▪▪▪▪▪▪▪▪▪▪□□□□▪□▪▪□□□▪▪▪▪	EAI3-IND	Indo-Oceanic-69

a As an extended explanation, one may refer to the example of case F, where profile 1 corresponding to MIT310 (LAM7-TUR lineage on the basis of spoligotyping), and profile 2 corresponding to MIT430 (T3-ETH lineage on the basis of spoligotyping), both correspond to the Euro American-40 sublineage. Indeed, profile 1 with deletion of the block 20–24 and profile 2 with deletion of the block 10–19 could indicate a possible common ancestor with all spacers in positions 10 to 24 being present. As indicated by our IS*6110*AD-typing data (see text), this hypothetical ancestor would be harboring a copy of IS*6110* between the spacers 19 and 20. Depending on the adjacent deletion located on the left or the right side of this IS*6110* would result in the 2 different spoligotype patterns observed here, i.e., profile 1 or 2. Hence, albeit phylogenetically very close, these 2 isolates would be classified as LAM7-TUR and T3-ETH, in the Brudey's classification scheme in SpolDB4.

b MIT; MIRU International Type according to the SITVITWEB database [5].

### 3. Phylogenetic inferences

Phylogenetic inferences were drawn using two applications: BioNumerics (version3.5, Applied Maths, Sint-Marteen-Latem, Belgium), and MrBayes3 (available through http://mrbayes.csit.fsu.edu/) [28]. BioNumerics (version3.5, Applied Maths, Sint-Marteen-Latem, Belgium) was used for phylogenetic reconstruction based on a “Minimum Spanning Tree” (MST) algorithm to draw MSTs on 7009 MTC patterns of the SITVIT2 database. For this purpose, allele strings were imported into a BioNumerics software package and a MST was created based on categorical and the priority rules (http://www.applied-maths.com/bionumerics/plugins/mlva.htm) with highest number of single locus variants (SLV's). Following the assumption that evolution required a minimum of evolutionary events and that all evolutionary states were present within the dataset studied, one could observe different taxonomic units that were clustered in the tree generated. In a MST, one considers that the internal nodes within a tree are part of the sample, and the branches illustrate agglomerations of variants around their common ancestor. MrBayes3 was used to infer phylogeny relationships among the 37 newly defined MIRU-VNTR lineages of *M. tuberculosis* sensu stricto using a bayesian approach that is particularly useful to reconfirm MST results [28].

**Figure 3:**
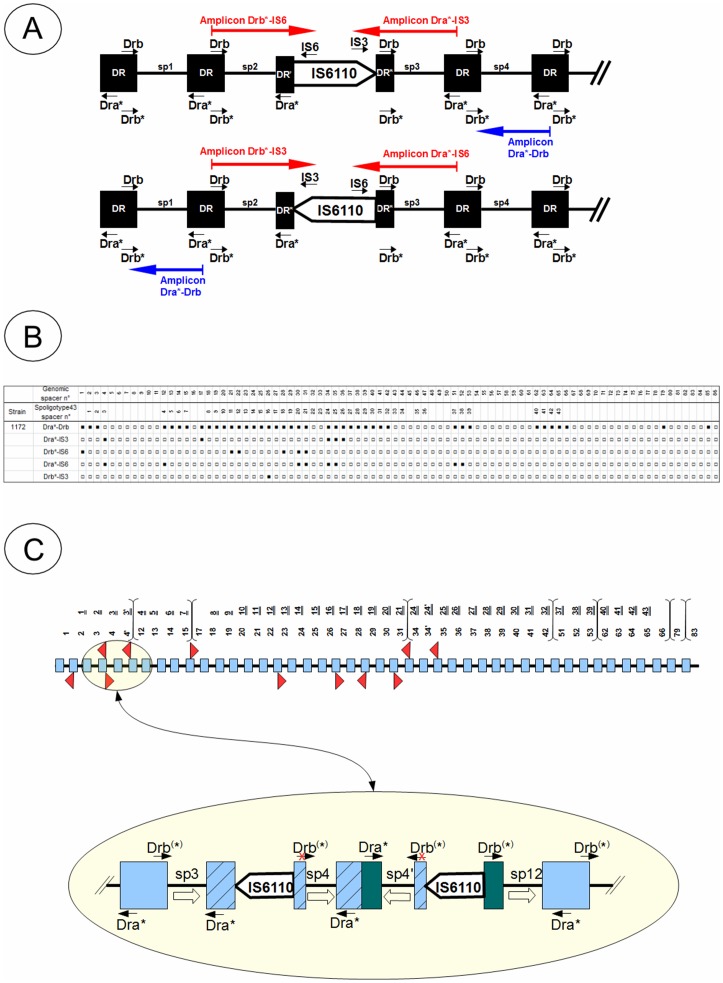
Some explanations on the technique of genotyping for the detection of IS*6110*. (A) An illustration for understanding the technique for detection of insertions of IS*6110* in the DR locus. (B) Result of genotyping of a strain (ID 1172) taken from a sample of 100 Mozambican strains. There are 5 distinct genotyping results with each of the primer sets shown; the 1^st^ line shows the classical spoligotyping while the remaining 4 lines show the detection of IS*6110* insertional events as detailed in the text. (C) Schematic representation of interpretation of the experiments shown in Figure 3B. Numbers underlined correspond to the numbering of the spacers in the 43-spacer spoligotyping format, while those not underlined correspond to the numbering of spacers according to their genomic position in the DR locus. The accolades mark the points of deletion of spacers.

### 4. Classification algorithm

To describe the classification algorithm, we must first explain the principle on which it is based. Take for example a MST done using 12-locus MIRUs on a set of 164 strains from Kerala, India (Figure S1). In this figure, the bigger circles surrounding the profile clusters are drawn according to the spoligotype-based lineage classification [5]. This tree also shows the 3 large phylogenetically relevant subdivisions based on *katG* and *gyrA* SNP polymorphism [8], which subdivides *M. tuberculosis* complex strains into three PGG groupings; PGG1 is considered to be evolutionarily older while PGG3 is the youngest which evolved from PGG2. Furthermore, ancestral strains are characterized by the presence of a specific deletion region (TbD1) as opposed to modern strains that are TbD1-deleted [9]. Superposition of these groupings suggests that PGG1 includes both ancestral (EAI) and modern (CAS, Beijing) lineages, while PGG2/3 include exclusively modern (Haarlem, LAM, T, and X) lineages [22], [23]. The MST shows the central nodes of EAI1-SOM, EAI3-IND, Beijing and CAS (CAS1-Delhi) lineages corresponding to respectively, MIT64, MIT69, MIT17 and MIT318 (Figure S1). This tree illustrates the fact that all lineage members congregate around a central node. Agglomeration includes all variants of a lineage while the central node represents the most common representative; hence it is the central node that generates the most variants within a given lineage.

**Figure 4:**
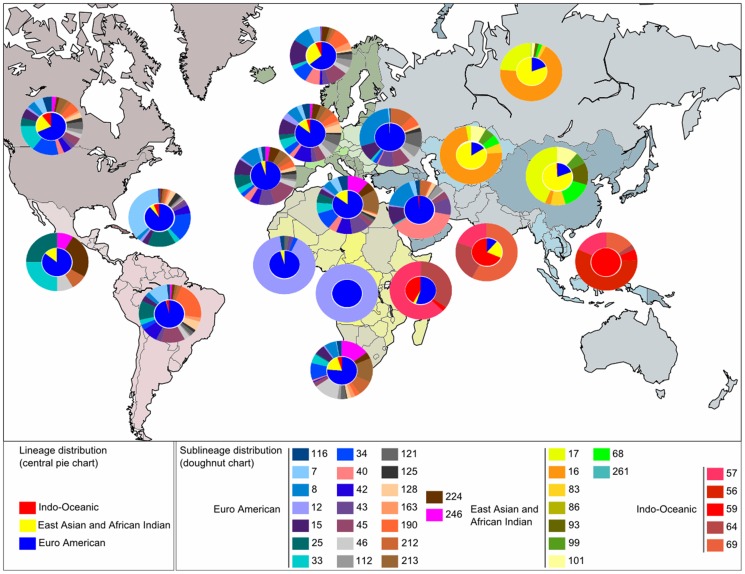
Global geographical distribution of the newly defined MIRU-VNTR lineages. In each subregion the distribution of the sublineages of the majority lineage is represented.

Thus classifying strains with MIRU-VNTRs in the present study amounted to identify (and define) all the central nodes as prototypes that in turn designated different phylogenetic groups; these were then submitted to a classification algorithm able to put each of them in one of these newly-defined families. The classification algorithm compared a pattern to all identified prototypes and retained the prototype(s) with which it shared most repeat values. If more than one prototype was retained, the algorithm calculated a cumulative Z-score (CZS) for each prototype retained to select the closest pattern defined by the lowest CZS. The CZS is defined as follows:
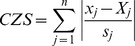



CZS is defined as the sum of the absolute value of 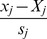
(which correspond to the Z-score) of each locus. 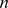 is the total number of distinct loci, 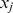 is the number of repeat of the _j_th loci, 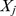 is the mean of repeat values of the _j_th loci and 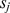 is the standard deviation of the _j_th loci). Note that for each MIRU-VNTR locus in a given MST agglomeration (i.e., a defined lineage), the distribution of number of repeats follows a Gaussian distribution; the 2 important parameters being the mean (*X*) and the standard deviation (*s*). For this purpose, the mean and standard deviation values of each of the 12 loci for MIRU based lineages were calculated. The resulting labeling, particularly for the newly defined lineages, was confronted both to spoligotyping- and LSP-based classification schemes [6], [22], [23]. A summary of reclassification of 176 MTC isolates from the MIRU-VNTRplus database (http://www.miru-vntrplus.org/MIRU/index.faces) by the MIRU based lineages versus spoligotyping- and LSP-based classification schemes is illustrated in Table S3. This kind of approach was particularly useful to label the 7009 reclassified strains originating from the SITVIT2 database while plotting the worldwide distribution of newly-described phylogenetical lineages.

**Figure 5:**
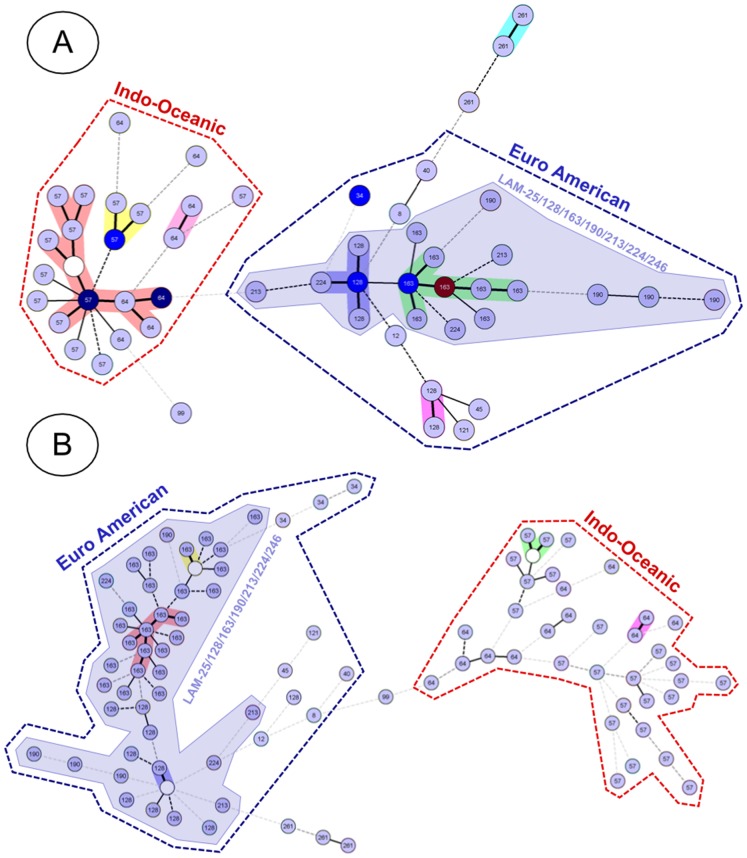
Two MST phylogenetic trees done with 95 Mozambican strains based on 12-loci MIRU-VNTRs (A), and 24-loci MIRU-VNTRs (B).

### 5. Geographical distribution of newly-described phylogenetical lineages

The worldwide distribution of newly-described phylogenetical lineages was studied both at the country level (3 letter country codes according to http://en.wikipedia.org/wiki/ISO_3166-1_alpha-3), as well as regional and sub-regional level according to the United Nations (http://unstats.un.org/unsd/methods/m49/m49regin.htm); Regions: AFRI (Africa), AMER (Americas), ASIA (Asia), EURO (Europe), and OCE (Oceania), subdivided in: E (Eastern), M (Middle), C (Central), N (Northern), S (Southern), SE (South-Eastern), and W (Western). In this classification scheme, CARIB (Caribbean) belongs to Americas, while Oceania is subdivided in 4 sub-regions, AUST (Australasia), MEL (Melanesia), MIC (Micronesia), and POLY (Polynesia). Note that Russia was attributed a new sub-region by itself (Northern Asia) instead of including it among the rest of Eastern Europe. It reflects its geographical localization as well as the similarity of specific TB genotypes circulating in Russia (a majority of Beijing genotypes) with those prevalent in Central, Eastern and South-Eastern Asia.

## Results and Discussion

### 1. Description of the lineages and sublineages identified

Phylogenetic inferences were drawn from 12-loci MIRU based MST constructed on all the 7009 MTC patterns taken from the SITVIT2 database (for which both spoligotyping and 12-loci MIRU-VNTR data were available; figure not shown since the resulting tree was over-crowded). From this tree, we came out with 7 major central nodes (or lineages) represented by the following MITs: 57, 17, 116, 934, 664, 49, 60 (Table 1). As summarized, lineages with node 57, 17, and 116 were subdivided into 37 sub-nodes as follows. Lineage 57 contained sub-nodes 57, 56, 59, 64, 69; lineage 17 included sub-nodes 17, 16, 83, 86, 93, 99, 101, 68, 261, the largest lineage 116 contained 23 sub-nodes: 116, 7, 8, 12, 15, 25, 33, 34, 40, 42, 43, 45, 46, 112, 121, 125, 128, 163, 190, 212, 213, 224, 246. A simplified MST showing the 7 major lineages and 41 sublineages is shown in Figure S2. The appropriate nomenclature for these lineages was proposed by comparing with previous classification schemes proposed in SpolDB4 [6] and by LSP-based classification [22], [23] in Table 1; see Table S3 for a re-classification of a set of well-characterized MTC isolates (n = 176 profiles) taken from the MIRU-VNTR*plus* online database (http://www.miru-vntrplus.org/MIRU/index.faces).

Interestingly, the MIRU-VNTR classification script run on the 7009 SITVIT2 dataset strains underlined a good overlap with the Gagneux's nomenclature (results not shown), which validates the names retained in Table 1. Thus, central-node 57 was named Indo-Oceanic, 17 as East Asian and African Indian, 116 as Euro-American, 934 as West African I, 664 as West African II, 49 as *M. bovis*, and 60 as *M. canettii* (Table 1 and Table S3). The sublineages were named by adding to the name the value of the central sub-node, e.g. Indo-Oceanic 57, Indo-Oceanic 56 etc. Note that we have combined 2 families described by Gagneux (East-Asian and East-African Indian), in a single large phylogenetic group called “East Asian-African Indian (EAAI)”. Indeed, during reclassification of the MIRU-VNTRplus profiles, the patterns of these two lineages were classified in the major node 17 (Table S3).

The above observation was corroborated by the reclassification of SITVIT strains where both CAS (East-African Indian) and Beijing (East-Asian) strains were reclassified in the node 17; nonetheless, both sublineages occupied distinct MIRU-based sub-nodes as summarized in Table 2. Thus starting from central node 17, Beijing consisted almost exclusively of sub-nodes East Asian-17, East Asian-16, East Asian-83, East Asian-86, East Asian-93, East Asian-99, East Asian-101, while CAS consisted mainly of East-African Indian-68 and East-African Indian-261. Further, CAS1-Delhi and CAS1-Kili profiles according to Brudey's classification are concentrated in East-African Indian-68 and in East-African Indian-261 sub-nodes, respectively. To validate the fact that the two phylogenetic groups of East Asian and East-African Indian in Gagneux's classification scheme form a single big group, we used a Bayesian tree with the 37 core MIRU-VNTR profiles retained within *M. tuberculosis* sensu stricto (Figure 1). In this figure, one can observe the three major phylogenetic groups (i) PGG1/TbD1+ (ii) PGG1/TbD1- and (iii) PGG2–3/TbD1- (shown in red, yellow, and blue colors) which clearly regroup Indo-Oceanic, East-Asian/East-African Indian, and Euro-American lineages, respectively. In the middle of this tree, East-African Indian-68 and East-African Indian-261 (the two CAS sublineages) share a central node with East-Asian sub-nodes 17, 16, 83, 86, 93, 99,101 (i.e., the Beijing sublineages), which corroborates the name “East Asian and African Indian (EAAI)” for this newly-defined large lineage represented by central-node 17.

In this MIRU classification, we note that the two lineages “Bov_4-caprae” and “AFRI1” as assigned by Brudey are compiled in a single phylogenetic group – “West African lineage II” (see Table 1, Table S3); this is an interesting observation knowing that AFRI1 shares with all of animal MTC pathogens (including BOV_4-caprae), a number of deletions (RD9, RD7, RD8, RD10), as well as a specific variation of 6 bp of the gene *pks* [9]. A unique strain (numbered 9550/00) from the MIRU-VNTRplus database and classified as West African II according to Gagneux's criteria, was reclassified in two distinct lineages: West African II and *M. bovis* (see Table S3). A MIRU-based MST tree drawn on 176 strains of the MIRU-VNTRplus database (Figure 2) showed that the three phylogenetic groups – West African I, West African II and *M. bovis* are phylogenetically close. Considering the fact that the oldest lineages are most distant from the Euro American lineage, the tree suggests that West African I and West African II lineage strains appeared before *M. bovis*. One may therefore speculate that the strain 9550/00 is a phylogenetic intermediate between these two lineages (West African II and *M. bovis*). It is further possible to make other analogies with Brudey's classification, especially for *M. tuberculosis* sensu stricto belonging to PGG1 group (Table 2), e.g., 77.14% of Indo-Oceanic-56 corresponds to EAI2-Manilla, 72.97% of Indo-Oceanic-69 corresponds to EAI3-IND, and 68.60% of Indo-Oceanic-64 corresponds to EAI1-SOM. However, it is more difficult to make similar correspondences among modern PGG2/3 lineages. These discrepancies between spoligotype based classification as described previously and the present insight using MIRU-VNTR based classification would need concerted efforts of wider research groups in coming years.

### 2. Differences observed between spoligotype and MIRU based classification schemes

As summarized briefly earlier and in Table 3, the MIRU-based classification superimposes quite well with that of Brudey for sublineages belonging to PGG1, nonetheless discrepancies do exist for PGG2/3 lineages; 2 broad categories can be cited regarding these discrepancies: (i) for cases A, B, C, D and E, where 2 patterns with a single spacer difference are classified in 2 separate lineages; (ii) cases F, G and H have blocks of missing spacers that are complementary among the 2 patterns. For 1^st^ category, one may consider case C – the pattern 1 (classified as CAS1-Delhi) has 3 blocks of spacers deleted (4 to 7, 23 to 34, and 37 to 38), while pattern 2 (classified as EAI5) differs from the first only by the presence of spacer 33. Both these patterns were classified as the East African-Indian-68 according to the MIRU-based classification scheme described in this paper. For 2^nd^ category (cases F, G and H), one may notice that blocks of spacers deleted in a given profile are contiguous to those verified in the other profile, e.g., pattern 1 in case F is characterized by a loss of spacers 20 to 24, 26 to 27 and 33 to 36 (classified as LAM7-TUR), while pattern 2 has 2 blocks of missing spacers; 10 to 19 and 33 to 36 (classified as T3-ETH).

It is important to recall that classical spoligotyping method which uses 43 spacers out of 104 reported spacers in tubercle bacilli [17], may not systematically reflect the succession and exact order of spacers on the genome, e.g., if the spacer block “20 to 24” of pattern 1 is indeed adjacent to the block “10 to 19” in pattern 2 (Table 3). We therefore thought it desirable to have a: (i) finer view of the DR locus using extended spoligotyping [15], [16], (ii) to detect IS*6110* insertions in the DR locus using methodology described earlier [18], [19], (iii) use IS*6110*AD-typing to investigate the role of IS*6110* insertional event(s) causing deletions in the MTC genome elsewhere than the DR locus. All these three techniques were used on a same set of 100 MTC isolates blindly sampled from an initial set of 445 clinical isolates studied in Mozambique [27]. The results obtained for selectected isolates are summarized in Figure 3 and Figure S3 for spacers 1 to 86 shown in sequential order, for the localization of IS*6110* insertions in the DR locus; and in Table S5 for IS*6110* AD-typing.

Regarding the demonstration of the IS*6110* in the DR locus (Figure 3A ), hybridization of a spacer by the primer sets (biot)DRa-IS3 or (biot)DRb-IS6 is positive evidence for IS*6110* insertion in the DR preceding the spacer in question in 5′→3′ direction, while with primer sets (biot)DRa-IS6 or (biot)DRb-IS3, it is an evidence for insertion in the direction 3′→5′. Nonetheless, asymmetrical insertion of IS*6110* in the DR can prevent the binding of one of the two primers and affect the amplification of the upstream or downstream spacer. Hence, we amplified the spacers both on the right and left of the DR repeats to evidence IS*6110* insertions; indeed these four pairs of primers are expected to produce an amplicon containing only a single spacer as shown in Figure 3A. The results obtained for the 86 extended spacers are summarized for a strain in Figure 3B (detailed results on 10 selected isolates from Mozambique are shown in Figure S3): 1^st^ line corresponds to use of classical spoligotyping primers DRa-Drb, while the 4 other lines correspond respectively to primer sets: (biot)DRa-IS3, (biot)DRb-IS6, (biot)DRa-IS6 and (biot)DRb-IS3, and are helpful to highlight the presence of IS*6110* element(s) in the DR locus. As shown in Figure 3B and Figure S3, the presence of IS*6110* often results in revelation of 1 or 2 adjacent spacers leading to 2 possible assumptions: (i) either there are several IS*6110* inserted into contiguous DR, or (ii) part of the amplicon carried by the IS*6110* had length variations (since transposable elements are know sometimes to carry pieces of genomic sequences; [29]). The results for strain 1172 reveal several IS*6110* in its DR locus, and they often occupy a position adjacent to the spacer blocks Figure 3B). Indeed, this strain presents several losses of spacer blocks: 4 to 11, 16, 32 to 33, 43 to 50, 54 to 61, 67 to 78, 80 to 84, and 86. This interpretation is schematized in Figure 3C, and underlines duplication of spacers in the 3′→5′ direction, e.g., for genomic positions 4 and 34, and corroborates previous reports [15], [30]. Thus, this DR locus would present 10 insertions of IS*6110* in the following locations: DR2 (located upstream of the spacer 2), DR4, DR12, DR17, DR23, DR27, DR29, DR31, DR34 and DR35. The presence of IS*6110* in the DR35 was already reported [31]. It is interesting to note that the insertions in DR4, DR12, DR17, DR31 and DR34 are adjacent to the absence of spacers 4 to 11, 16, and 32 to 33.

In the context of adjacent deletions, the potential role of homologous recombination between two IS*6110* insertions was underlined for the RvD2 deletion and disruption of the plcD gene in *M. tuberculosis* [32]. Indeed, the IS*6110*-associated deletion hypervariability is today considered an important driving force in *M. tuberculosis* genome evolution [33]. As illustrated in Table S6, several Regions of Difference (RD) are reportedly located next to IS*6110*, e.g., RD152, RD207, RD5, RD11, RD14, MiD2 [34], [35], [36]. To determine whether the IS*6110* was involved in genetic recombination that may cause adjacent deletions [20], we applied IS*6110* AD-typing to selected strains from Mozambique and *M. tuberculosis* H37Rv. The results obtained underlined deletions adjacent to IS*6110* insertions (Table S5). Unexpectedly, we also observed deletions in *M. tuberculosis* H37Rv not reported in the original H37Rv sequence on the NCBI server; these deletions probably occurred during successive subcultures of the type strain in our laboratory for almost 18 years. This observation was indirectly corroborated by the fact that we also observed an additional deletion in the spoligotype pattern of this H37Rv strain (Figure S3); indeed the strain in our case lost spacer 15 (in addition to the characteristic H37Rv pattern defined only by the absence of spacers 20 to 21 and 33 to 36), although its MIRU-VNTR pattern remained unchanged. Considering that the test isolates were not repeatedly subcultured, we presume that similar deletions did not occur during the time of the study.

One may postulate that the high IS*6110* copy number in the H37Rv genome (16 copies) conferred a high mutation rate to the DR locus, since the latter is know to be an IS*6110* preferential locus (*ipl* ; [21]). However, mechanisms other than IS*6110* insertion have been suggested to cause the loss of spacers in the DR locus – which is a member of the Clustered Regularly Interspaced Short Palindromic Repeats (CRISPR) – such as homologous recombination between DR [37] or IS*6110* [33], and slippage during DNA replication [38]. In a recent study, different spoligotypes observed among epidemiologically related strains were attributed to the loss of spacer blocks due to recombination between DRs, an event favored by the formation of a secondary structure involving two IS*6110* in opposite orientation [31], an explanation that argues in favor of more complex and interlinked way of MTC evolution involving 2 or more mechanisms simultaneously. In conclusion, insertion sequences undoubtedly induce adjacent deletions [20], and no matter the mechanism, the fact that IS*6110* are observed next to deleted spacers on the DR locus underlines their active involvement in DR evolution by loss of spacers.

In conclusion, the discrepancies observed between spoligotype and MIRU based classification schemes in the cases cited above underline that MIRU-based classification tends to group MTC isolates that are phylogenetically close or almost similar albeit they might appear distant if only judged based on their spoligotyping patterns. For example, going back to the Table 3 (case F), where the profile 1 presents the deletion of the block 20–24 (in classical 43-spacer numbering), and profile 2 a deletion of the block 10–19. If these 2 profiles shared a common ancestor, it would have all the spacers in positions 10 to 24 present, and in addition would harbor a copy of IS*6110* in the DR located between the spacers 19 and 20. Thus depending on the adjacent deletion located on the left or the right side of this IS*6110* would result in totally different spoligotype patterns that would be classified in 2 distinct sublineages according to SpolDB4 classification (classified as LAM7-TUR and T3-ETH, respectively), albeit phylogenetically very close. Hence, the MIRU-based classification scheme that groups these 2 spoligotypes together is appropriate.

The Euro American phylogenetic group of Gagneux that groups TbD1-/PGG2/3 spoligotype-defined lineages (Haarlem, LAM, X, S, and T), as well as a wide range of unclassified spoligotype profiles in the recent SITVITWEB version of the international database [5], is characterized by the presence of a high number of IS*6110* copies. The large copy number of IS*6110* in these modern strains produces many variations in the DR locus, making it difficult to study their evolution uniquely on the basis of their spoligotype profile. Further, asymmetrical IS*6110* insertional events could also lead to 2 patterns differing by a single spacer change [18], [19], and falsely lead to their inclusion in two different lineages based on certain SpolDB4 lineages. On the contrary, TbD1+/PGG1 ancestral EAI lineage harbors little or no IS*6110*, which explains a good concordance between spoligotype and MIRU-based classification schemes.

### 3. Global geographical distribution of new MIRU-VNTR lineages

#### 3.1. The global distribution map of the MIRU-VNTR lineages

The global geographical distribution of the newly defined MIRU-VNTR lineages is summarized in Figure 4. The map drawn illustrates the information available in the SITVIT2 database for the 6800 MTC isolates recognized as *M. tuberculosis* sensu stricto. The figure shows pie charts with two circles – the inner circle shows the three most predominant newly-described lineages, i.e., Indo Oceanic, East Asian and African Indian (EAAI), Euro American, whereas the outer circle shows the sublineages belonging to uniquely the most predominant of the three lineages (please refer to the color scheme shown in the legend to Figure 4). The exception being the region corresponding to East Africa for which both the lineages Euro American and Indo Oceanic were almost equally represented (almost 50% of strains). Note that we chose to illustrate the distribution of Indo Oceanic sublineages since this lineage followed a distribution gradient from South-East Asia to East Africa for regions bordering the Indian Ocean (see below). Thus the outer circles show the distribution of following sublineages: Indo Oceanic in AFRI-E, ASIA-SE and ASIA-S; East Asian and African Indian (EAAI) in AFRI-E/ASIA-E, ASIA-C and ASIA-N; Euro American in all other subregions essentially in Europe and Americas. Briefly, one may conclude that the Indo Oceanic lineage is widely represented in AFRI-E (42.11%), ASIA-S (68.31%), and ASIA-SE (100%); East Asian and African Indian (EAAI) in ASIA-C (84.44%), ASIA-E (80%), and ASIA-N (80.59%); and Euro American lineage in all other sub-regions, e.g., it represents 68.31% of TB cases listed in AMER-N, 96.15% in AMER-S, 89% in CARI, 64.25% in EURO-N, 85.71% in EURO-W, 94.53% in EURO-S, 99.26% in EURO-E, 98.43% in ASIA-W; 95.45% in AFRI-W, and 100% in AFRI-M.

#### 3.2. Out of Africa scenario: Indo Oceanic lineage

Seeing the phylogeographical specificity of the Indo Oceanic lineage (which according to the Bayesian tree is the more ancient among the three major phylogenetic groups) for regions bordering the Indian Ocean, it seems to have originated on the east coast of Africa. Indeed, the Indo Oceanic-57 sublineage, found in strong proportion in East Africa followed by India and South-East Asia could be considered as the central node of the Indo Oceanic lineage (Figure 1). According to this tree, Indo Oceanic-69 (prevalent in India) and Indo Oceanic-56 sublineages (predominant in South-East Asia) share a close common ancestor. According to the length of tree branches, Indo Oceanic-69 sublineage apparently diverged from this common ancestor before the Indo Oceanic-56 lineage (Figure 1). These observations suggest a human migration from the East of Africa to South-East Asia (Pacific Islands) via India. Further, the global geographical distribution of *M. tuberculosis* (lineages) sensu stricto underlines that this migration would not have affected the North of the Middle East. In this context, it might be worthwhile to mention that almost all the strains belonging to the Indo Oceanic lineage in Middle East are concentrated in its south, specifically Saudi Arabia. This pattern of *M. tuberculosis* evolution and migration through its human host is corroborated by studies based on human mitochondrial DNA (mtDNA) showing a first migration route out of Horn of Africa [39]; the migrants successively joined the Arabian coast and Persia [39], [40], followed by India and Thailand, Indonesia and Australia [39], [41], [42] – a migration dated back to an interval ranging from 80,000 to 60,000 years.

#### 3.3. The Asian continent, place for the East Asian and African Indian (EAAI) lineage expansion

In the Bayesian tree (Figure 1), the central place is occupied by the East Asian and African Indian (EAAI) lineage characterized by TbD1-/PGG1 strains that predominate in Asia (Figure 4). The distribution of sublineages showed a high proportion of East Asian-17 sublineage in ASIA-E region (43.70%), followed by East Asian-16 in ASIA-C (74.68%) and ASIA-N (68.4%). Considering the two types of Beijing lineages in Asia; a 1^st^ type being characterized by the presence of a NTF region without IS*6110* insertion while the 2^nd^ type presents an IS*6110* in the NTF locus [43] – the former could correspond to East Asian-16 sublineage while the latter would correspond to East Asia-17. Indeed, a study based on human phylogeography hypothesized that the 1^st^ type emerged in the upper Paleolithic period in Central Asia among the NRY K-M9 haplogroup coming from the Middle East [43]. The geographical location of different descendant haplogroups suggests that the migration route would then concern the North East (to Siberia) and the South East (northern China). This Paleolithic Beijing which prevails in central Asia and north Asia, superimposes with the geographical distribution of the East Asian-16 sublineage. Similarly, the 2^nd^ Beijing type would have emerged in the Neolithic period among Proto-Sino-Tibetan farmers in East Asia (Haplogroup O-M214/M122) followed by its spread to the rest of East Asia [43], which coincides well with the predominance of East Asian-17 sublineage over the same geographical area. Further studies will be needed to investigate if both Beijing differentiated by Mokrousov et al. [43] are blended with East Asia-16 and East Asia-17 sublineages as suggested by our distribution map (Figure 4).

#### 3.4. The Euro American lineage was probably first spread to Europe through several human migrations from Middle East: the Asia-to-Africa back migration theory

Considering that the Indo-Oceanic (EAI in SpolDB4) lineage is the most ancestral [9], the Euro American lineage is the latest to emerge according to the Bayesian tree (Figure 1). On the map shown in Figure 4, this lineage is predominant in Europe and America which largely justifies its name. According to the Bayesian tree, Euro-American-40 was the first to emerge among subfamilies belonging to the PGG2/3 group (Figure 1); considering that it is also highly predominant in western Asia (with 37.6% of the PGG2/3 strains, Figure 4), we suggest that the ancestor of all modern MTC strains probably originated in this sub-region. Furthermore, although the distribution of Euro American sublineages in various regions is quite heterogeneous; this is not the same for middle and western Africa, where the Euro American-12 sublineage predominates (100% and 90.48%, respectively, of all modern strains). To better understand how modern strains are found in Africa in such proportions, one may refer to the trajectory of R1b haplogroup (Y-chromosome). R1b is most frequently found in western Europe, parts of central Eurasia and in parts of sub-Saharan and central Africa, e.g., around Chad and Cameroon (http://en.wikipedia.org/wiki/Haplogroup_R1b_(Y-DNA)#Origin_and_dispersal). The point of origin of this haplogroup is thought to lie in Eurasia, most likely in western Asia [44].

We also attempted to explain the present distribution gradient of the Euro American lineage on the basis of an Asia-to-Africa back migration theory; indeed, Cruciani et al. [25] underlined an unusual Asia-to-Africa back migration at Neolithic time through the study of Y-chromosome haplogroups. In an attempt to test this hypothesis (Asia-to-Africa back migration) with the information contained in the 12 loci MIRU-VNTR of *M. tuberculosis* strains, we classified 154 published strains from the north west of Iran [26] with our new classification algorithm. The first three lineages that predominate in this region are Euro American-212 sublineage with 22.8%, *M. bovis* lineage with 21.43% and Euro American-121 sublineage with 11.69% (data not shown). Considering that Euro American-121 sublineage contains African strains (like the Euro American-12 sublineage, Table S3), the reclassification of strains taken from the MIRU-VNTR*plus* database further underlined the fact that Euro American-121 sublineage included strains belonging to the Uganda II and Ghana spoligotype families, while Euro American-12 sublineage included mainly strains of the Cameroon family (Table S3). The phylogenetic tree in Figure 2 shows that Euro American-121 and Euro American-12 sublineages are close and that the former would be older than the latter. The high prevalence of Euro-American-121 sublineage strains in Iran, and that of Euro-American-12 sublineage strains in central and western Africa also confirms the assumption regarding Asia-to-Africa back migration of the Euro American lineage.

In the sample of MTC isolates from the north west of Iran, we observed a high proportion of Euro American-212; reclassification of MIRU-VNTR*plus* strains (Table S3) showed that this sublineage exclusively corresponds to the S sublineage in SpolDB4 [6] (an observation also confirmed by classification of SITVIT strains), with reported phylogeographical specificity to Sicily and Sardinia [45]. The high prevalence of this lineage in the north west of Iran allows us to speculate that it may have originated in the Middle East and reached the mediterranean coast by migrants at Neolithic period, harboring the R1b haplogroup [44]. It is therefore clear that the prevalence of the Euro American lineage in Europe and America cannot be explained solely on the basis of recent European colonization but also due to first human migrations in America through Bering Strait from Asia about 20,000 years ago (atlas of human journey: https://genographic.nationalgeographic.com/genographic/lan/en/atlas.html). We note that outside Asia, East African Indian-68 sublineage is predominant among modern TbD1-/PGG1 strains in some subregions, e.g., Northern Europe (37.71%), Western Europe (31.51%) and Northern Africa while East-Asian-17 is predominant in North America (43.13% among EAAI) strains and further concentrates most of the EAAI strains in central America.

#### 3.5. Identification of two major phylogenetic groups among Euro American lineage

The Bayesian tree in Figure 1 shows many sub-nodes each with a distinct sublineage; nonetheless 2 sub-nodes are slightly more distal and lead to secondary branching leading to two additional phylogenetic sub-branches within the Euro American lineage: (i) a 1^st^ group with sublineages 45, 43, 42 (ii) a 2^nd^ group with sublineages 213, 190, 246, 25, 163, 224, 128. To name these two sub-groups, we did an analogy with the SpolDB4 lineages as updated recently in the SITVITWEB [5]. We observed that 92% of Haarlem lineage strains correspond to the 1^st^ group, hence it was renamed as *Haarlem-42/43/45*. Further, 74.33% of LAM and 100% T5-RUS1 strains were found in the 2^nd^ group; considering that T5-RUS1 was recently reclassified as LAM on the basis of specific SNPs [14], [46], [47], the 2^nd^ group was renamed as *LAM-25/128/163/190/213/224/246*. The worldwide distribution of these 2 sub-groups is summarized in Table S7; the *Haarlem-42/43/45* phylogenetic group is well represented in Europe, mainly in the south of Europe and South America, as well as in North Africa; whereas the *LAM-25/128/163/190/213/224/246* phylogenetic group – subdivided in subgroups A (*LAM-190/213/246*) and B (*LAM-25/128/163/224*) – is well distributed everywhere in Europe, Africa and America, except in EURO-S, ASIA-W, AFRI-M, and AFRI-W. The fact that Euro American-213 (at the base of the sub-node and supposedly more ancient than other terminal sublineages in the 2^nd^ group) is prevalent in the North of Africa may suggest its emergence in this subregion.

#### 3.6. Tentative identification of LAM strains harboring the RD^rio^ deletion among the *LAM-25/128/163/190/213/224/246* phylogenetic group

To answer this question, we reclassified 190 published strains from Rio de Janeiro [24]. As summarized in Table S4, the study sample contained wild-type strains (n = 90), strains with RD^rio^ deletion (n = 93), and international reference strains harboring the RD^rio^ deletion (n = 7). The results obtained showed that of the 100 strains with RD^rio^ deletion: (i) the majority (95%) belong to the *2^nd^* phylogenetic group shown above (sub-group B *LAM-25/128/163/224*) (ii) A minority (3%) belonged to sub-group A *LAM-190/213/246*; (iii) and 2 were not part of either of the LAM phylogenetic groups. On the contrary, the distribution of the wild-type strains (n = 90) was different with a majority of the sub-group A strains as follows: (i) the majority (70/90 or 77.8%) belonged to the sub-group A *LAM-190/213/246*; (ii) A minority (11/90 or 12.2%) belonged to the sub-group B (*LAM-25/128/163/224*); (iii) and 9 were not part of either of the LAM phylogenetic groups. These results may be further interpreted based on the Figure 1 , where LAM sub-groups A (Euro American sublineages *190/213*/246) and B (Auro American sublineages *25/128/163/224*) appear to have apparently diverged from a common LAM ancestor that they share in common with the sublineage 213. One may therefore conclude that the LAM ancestor initially had an intact RD^rio^ region, from which diverged Euro American-213 and the predecessor of Euro American-190 and -246 sublineages (various group A strains that are characterized by an intact RD^rio^ region). Later, the loss of the RD^rio^ region constituted the phylogenetic sub-group B (*LAM-25/128/163/224*). As summarized in Table S7, the subgroup A which is found in Southern Africa (78.27%) and North of Africa (68%), is more ancestral than the subgroup B which is well represented in Caribbean (89.5%) and south & west Europe (58.8% and 60.3% respectively).

#### 3.7. The ability of 12-loci versus 24-loci MIRU-VNTRs to discriminate MTC sublineages

To answer this question, we constructed 2 MST phylogenetic trees with 95 strains of Mozambique (Figure 5), which essentially contained 2 main lineages – Indo-Oceanic (42.1%) and Euro American (54.7%). Irrespective of the typing format used (12-loci, Figure 5A vs. 24-loci, Figure 5B), none of the trees showed a strong link between these two main lineages. Almost the totality of Euro American strains (84.6%) belonged to the LAM phylogenetic sub-group B (essentially sublineages Euro American-163 and Euro American-128). Regardless of the typing format used, the trees showed the same two big clusters (even though the tree made with 24-loci had much more ramifications). We therefore conclude that 12-loci format is sufficient to discriminate the present MIRU-VNTR based MTC lineages.

### 4. Concluding remarks

This paper provides new information on the MTC genotypic polymorphism based on widely used markers, i.e., IS*6110*, the DR locus, the LSPs and MIRU-VNTR minisatellites. The genotypic classification of MTC was until now based on SNPs [48], LSPs [22], [23], and spoligotyping [5], [6]. Although spoligotyping-based classification was more discriminative than the LSP-based classification, it was recently singled out as subject to convergent evolution of the DR locus [14]. In this regard, although the MIRU-VNTR typing has been massively used for MTC molecular typing in recent years, its use for purely phylogenetical classification of MTC was not investigated at a large scale.

By using the MST method in conjunction with a Bayesian approach in this investigation, we describe a 12-loci MIRU scheme for MTC classification. This study also showed evidence for the satisfactory ability of 12-loci MIRUs to discriminate MTC sublineages versus 24-loci format. In light of the information provided herein, the genotypic classification of MTC lineages based until now on spoligotyping and LSPs is now rendered more accurate thanks to MIRU-VNTR minisatellites. We therefore recommend that future investigations using MIRU-based typing of *M. tuberculosis* refer to the present classification for lineage attribution in addition to existing spoligotyping and/or MIRU based systems. Indeed, seeing the complex sublineage names in the present nomenclature, a time of adaptation might be necessary for many of the users (or databases), already providing with a lineage attribution.

Comparison of this new classification to that of Gagneux, demonstrated that (i) the Indo-Oceanic lineage is divided into five phylogenetic subgroups, (ii) the East- African Indian and East Asian lineages form one large group which is subdivided into nine phylogenetic subgroups, (ii) the Euro American lineage contains twenty-three subgroups, and that (ii) the West African II lineage includes the BOV_4-CAPRAE sublineage [6]. In general, phylogenetic PGG1 sublineages of Brudey, find a match in this new classification, which is not always the case for PGG2/3 spoligotype sublineages [6]. For instance, both the Haarlem and LAM groups and subgroups were correctly identified in our new classification scheme. Furthermore, within the LAM family, RD(Rio) sublineages could be identified. Often, discrepancies observed between the 12-loci MIRU based classification and the spoligotyping methods were resolved, since MLVA-based classification tends to group MTC isolates that are phylogenetically close or almost similar albeit they sometimes appeared distant if judged solely based on their spoligotyping patterns. Indeed, these supposedly distant spoligotype patterns arose due to IS*6110* insertional events that could be implicated in loss of DR locus spacers. Thus, our results also underlined the role of transposable elements in chromosomal rearrangements, since there is a direct link between the large number of IS*6110* elements found in the DR locus and deletions of DR spacers causing the bulk of polymorphism occurring in this genomic region. Hence even if much of the IS*6110* transpositional events may not be traced as being directly involved in convergent evolution of MTC strains, a fair portion of convergence leading to the currently observed bias in phylogenetic classification of strains may be traced back to the presence of IS*6110*. Besides, our results suggest that IS*6110* may be implied in a fraction of the LSP deletions, and may therefore play a role in the high level of MTC genomic plasticity conferring its adaptation to a wide variety of hosts and environment.

In our opinion, MTC strains having a high number of IS*6110* elements such as those belonging to the Euro American lineage, would highly benefit from MIRU-VNTR typing to assign a phylogenetic position translating evolutionary reality. The novel MIRU-VNTR based classification scheme presented in the present investigation seems to be a good alternative to support future phylogenetic and epidemiologic studies. Considering its cost effectiveness and simplicity, the MIRU-VNTR typing in conjunction with the present MTC classification scheme is equally appropriate both for developed and emerging nations concerned by tuberculosis. Last but not least, the results presented herein on a first worldwide phylogeographic snapshot of MTC diversity and evolution as judged by their MIRU-VNTR profiles shed new light on the evolutionary history of the pathogen in relation to the history of peopling and human migration.

## Supporting Information

Figure S1 MIRU-based minimum spanning tree (MST) constructed on 164 *M. tuberculosis* isolates from Kerala, India (unpublished results, see acknowledgments section for origin of data). This tree was made using the BioNumerics software, and illustrates the fact that all lineage members congregate around a central node. The tree illustrates MIRU based subdivisions concomitantly with other phylogenetically relevant markers: (i) *katG-gyrA* polymorphism based three principal genetic groups (PGG); (ii) spoligotype-based lineages; and (iii) presence of a specific deletion region (TbD1).
(PDF)

Figure S2 **A Minimum Spanning Tree (MST) constructed on MIRU-VNTR prototype MITs defining the newly described sublineages.** Please refer to the text for further details.
(PDF)

Figure S3 **Result of genotyping on 10 MTC strains** (**selected from a sample of 100 Mozambican strains**)**.** There are 5 distinct genotyping results with each of the primer sets shown; the 1^st^ line shows the classical spoligotyping while the remaining 4 lines show the detection of IS*6110* insertional events as detailed in the text.
(PDF)

Table S1 **Description of primers used for amplification of sequences adjacent to IS** ***6110*** **present in** ***M. tuberculosis*** **H37Rv.** IR-r (Inverted Repeat Right) refers to the inverted repeat sequence that frames the IS*6110* in the 5′ side. IR-l (Inverted Repeat Left) refers to the inverted repeat sequence that frames the IS*6110* in the 3′ side. The amplicon name comprises the ID of the IS*6110* followed by the symbol “–“ then the letter “r” (right) for amplicon located in the 5′ or “l” (left) for the 3′ side.
(PDF)

Table S2 (A) Protocol for the MIX preparation of each IS*6110*AD-typing multiplex. (B) Program cycles used. The process from second to fourth cycle (2*, 3*, 4*) was repeated 35 times.
(PDF)

Table S3 **Reclassification of 176 profiles taken from the MIRU-VNTR** ***plus*** **database (** http://www.miru-vntrplus.org/MIRU/index.faces **).**
(PDF)

Table S4 **Reclassification of 190 published strains** [**24**] **.** This collection gathers data from Rio de Janeiro with RD^rio^ deletion (n = 93 strains), international strains with RD^rio^ deletion (n = 7), and other Rio de Janeiro strains which contained the RD^rio^ sequence (n = 90).
(PDF)

Table S5 **Results of IS** ***6110*** **AD-typing performed on 10 Mozambican strains.** Filled square symbolize intact region, empty squares symbolize regions deleted. An asterisk (*) is added when the region size has about 50 bp less than the expected size.
(PDF)

Table S6 **Description of selected regions of difference (RD) located in an adjacent position to an IS** ***6110*** **.** The first column gives the name of the locus of the two transposases of the concerned IS*6110*. The second column gives the RD that is adjacent to IS*6110* insertion. The third column lists of position of gene(s) involved in the deletion.
(PDF)

Table S7 **Distribution of phylogenetic groups in the various sub-regions of the world.** Percentage of a given group among PGG2/3 isolate is reported in each subregion. (A) Distribution of *Haarlem-42/43/45* (B) Distribution of *LAM-25/128/163/190/213/224/246* group and these two subgroups *LAM-190/213/246* (subgroup A) and *LAM-25/128/163/224* (subgroup B).
(PDF)
