# Supplementary figures and images for: MLVA Based Classification of Mycobacterium tuberculosis Complex Lineages for a Robust Phylogeographic Snapshot of Its Worldwide Molecular Diversity
Source: PLoS One. 2012 Sep 11;7(9):e41991. doi: 10.1371/journal.pone.0041991 (PMC3439451; doi:10.1371/journal.pone.0041991)

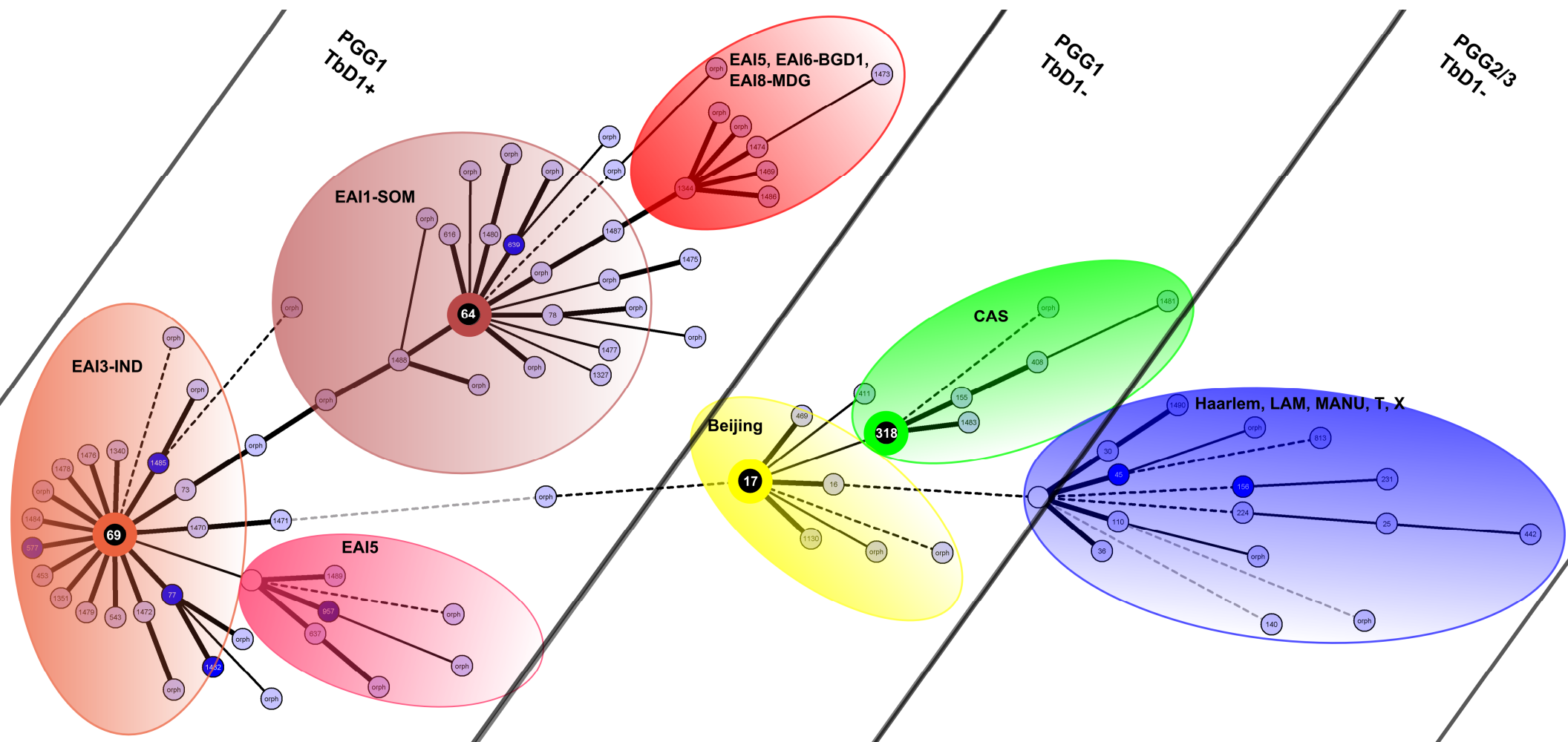

Supplement: Figure S1 — MIRU-based minimum spanning tree (MST) constructed on 164 M. tuberculosis isolates from Kerala, India (unpublished results, see acknowledgments section for origin of data). This tree was made using the BioNumerics software, and illustrates the fact that all lineage members congregate around a central node. The tree illustrates MIRU based subdivisions concomitantly with other phylogenetically relevant markers: (i) katG-gyrA polymorphism based three principal genetic groups (PGG); (ii) spoligotype-based lineages; and (iii) presence of a specific deletion region (TbD1). (PDF) [file pone.0041991.s001.pdf]

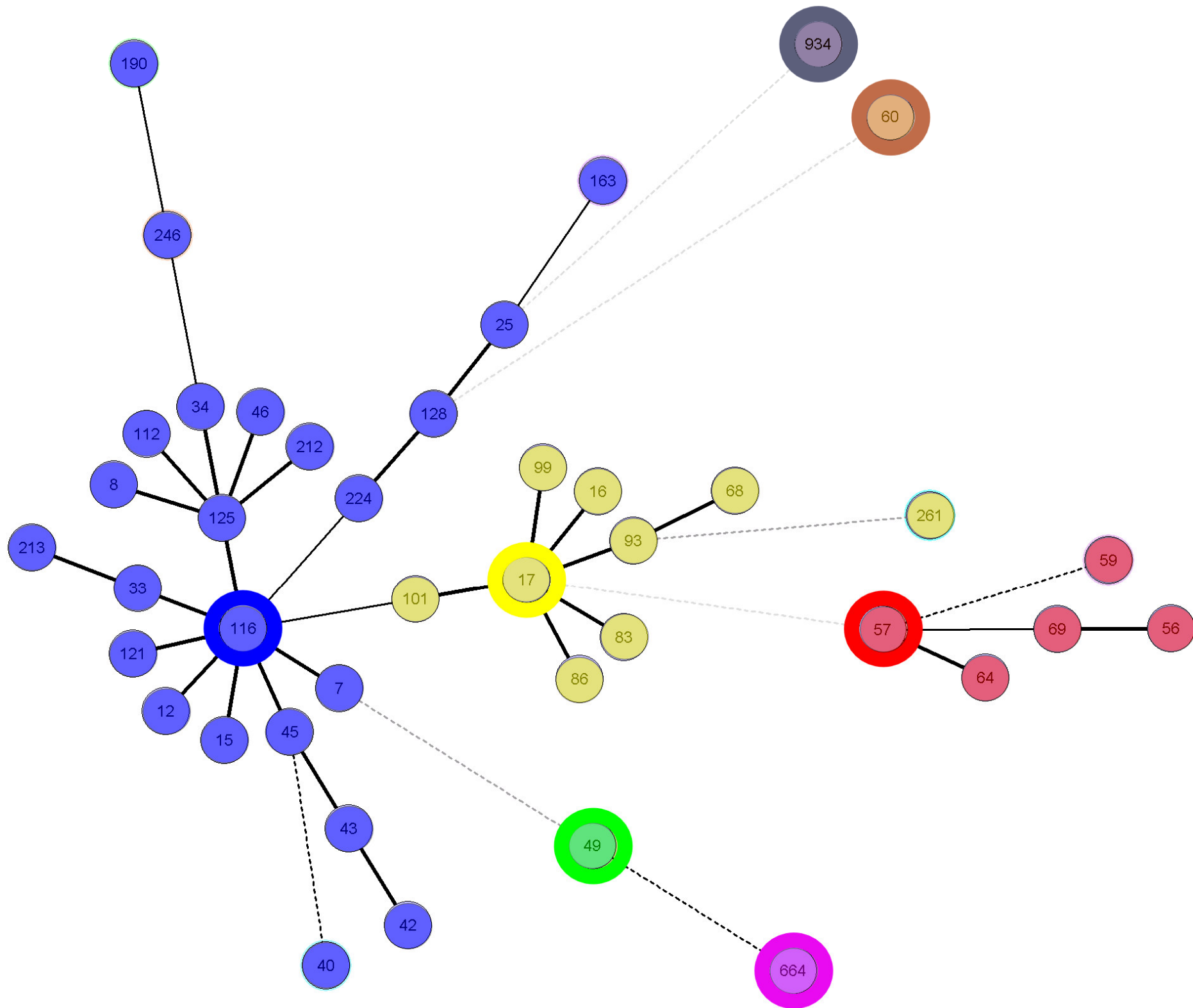

Supplement: Figure S2 — A Minimum Spanning Tree (MST) constructed on MIRU-VNTR prototype MITs defining the newly described sublineages. Please refer to the text for further details. (PDF) [file pone.0041991.s002.pdf]
