# Supplementary material for: MLVA Based Classification of Mycobacterium tuberculosis Complex Lineages for a Robust Phylogeographic Snapshot of Its Worldwide Molecular Diversity
Source: PLoS One. 2012 Sep 11;7(9):e41991. doi: 10.1371/journal.pone.0041991 (PMC3439451; doi:10.1371/journal.pone.0041991)
Supplement: Table S1 — Description of primers used for amplification of sequences adjacent to IS 6110 present in M. tuberculosis H37Rv. IR-r (Inverted Repeat Right) refers to the inverted repeat sequence that frames the IS6110 in the 5′ side. IR-l (Inverted Repeat Left) refers to the inverted repeat sequence that frames the IS6110 in the 3′ side. The amplicon name comprises the ID of the IS6110 followed by the symbol “–“ then the letter “r” (right) for amplicon located in the 5′ or “l” (left) for the 3′ side. (PDF) [file pone.0041991.s004.pdf]

<u>Supplemental Table S1.</u> Description of primers used for amplification of sequences adjacent to IS*6110* present in H37Rv. IR-r (Inverted Repeat Right) refers to the inverted repeat sequence that frames the IS*6110* in the 5' side. IR-l (Inverted Repeat Left) refers to the inverted repeat sequence that frames the IS*6110* in the 3' side. The amplicon name comprises the ID of the IS*6110* followed by the symbol "-" then the letter "r" (right) for amplicon located in the 5'or "l "(left) for the 3' side.

	ID IS*6110*	IS*6110* position (pb)		Amplicon name	Forward Primer 5'-3' (FP)	FP position (bp)	FP Tm	Reverse Primer 5'-3' (RP)	RP position (bp)	RP Tm	Amplicon length (bp)
**Multiplex 1**	1	IR-r start (pb)	889022	**1-r**	CGCCGACGATCGCAA	888041	62.71	TGAGGTTGGGCCGGT	888997	62.86	956
		IR-l end (pb)	890374	**1-l**	CCAACTCGGTGAGAAGACC	890455	62.24	CCTTTGGTAGTAGGCCTGG	891310	61.68	855
	2	IR-r start (pb)	1541953	**2-r**	GAAGCCCAGCAGATCCTG	1541187	62.47	GGCCTTGGCGAATTCTCA	1541919	62.71	732
		IR-l end (pb)	1543305	**2-l**	GACAAACGACCTCCCAGAC	1543360	62.54	CCACATAGTTGAGATCGAAGG	1544015	60.25	655
	3	IR-r start (pb)	1987704	**3-r**	TTGAACGTTGACGTCATGTC	1987052	61.18	TCAAACAGTCAGCCGATCC	1987607	62.26	555
**Multiplex 2**		IR-l end (pb)	1989056	**3-l**	CCGTGATGGGCTTTGTTAC	1989252	61.33	CACTCCAAGTCACAGCTCA	1989695	61.81	443
	4	IR-r start (pb)	1996102	**4-r**	CATGCAGCAATGCGAACTC	1995739	62.70	CCGACCTTTGTCCTGTTGA	1996094	62.12	355
		IR-l end (pb)	1997454	**4-l**	GCAACCGACGATTGATCTGA	1997620	62.89	AATGTTGAGGCGTTTCTGC	1997875	61.56	255
	5	IR-r start (pb)	2365415	**5-r**	TTGCTATGGCCGCTTACTG	2365127	62.70	AAAGTGCTCGACTTGGTGG	2365287	62.78	160
		IR-l end (pb)	2366767	**5-l**	CACGTTCCGTATTCGGTGTA	2366916	62.25	ACTCCCTCTTGAACGCTTAG	2366971	61.58	55
**Multiplex 3**	6	IR-r start (pb)	2430118	**6-r**	GATGGTCGCGTCGAGAAG	2429131	62.83	ATATTGAGTCCGGTTTGAGCC	2430086	62.54	955
		IR-l end (pb)	2431470	**6-l**	CTTCCCGAGACTGCGATTC	2431475	62.73	CGTCGACATACACCGTCAG	2432312	62.68	837
	7	IR-r start (pb)	2550015	**7-r**	CGGTAGTCGTCGACAGTTT	2549196	61.91	AGACTGACGACCCGATGAT	2549949	62.67	753
		IR-l end (pb)	2551367	**7-l**	GTTGGAAAGTGAGGCCTCC	2551539	62.88	ATGCAGCTTGACGATCACC	2552194	62.99	655
	8	IR-r start (pb)	2635578	**8-r**	TTGATGTTGCCCGAGTTCC	2634921	62.87	GCGACACGAACTTCGGTAA	2635476	62.85	555
**Multiplex 4**		IR-l end (pb)	2636930	**8-l**	ACAACTCGGTTTCTGCAGG	2637336	62.78	GGTATCTCCAACTCGGGTG	2637791	61.73	455
	9	IR-r start (pb)	2784616	**9-r**	CCTAAGCCGATATGGGCAG	2784121	62.26	TATCACCGTCAATTGCTGGG	2784476	62.59	355
		IR-l end (pb)	2785968	**9-l**	AATGAAGCGGTAACTGTGGAT	2786132	62.14	CAGTAACTATCGCGCCGAG	2786387	62.83	255
	10	IR-r start (pb)	2972110	**10-r**	TTAGGCTCCATGGAGCCTAA	2971853	62.60	TAAACCATCCCGGTAGCCA	2972008	62.96	155
		IR-l end (pb)	2973462	**10-l**	GTAGTCGTTGACTGATCTCGC	2973609	62.85	AGAACCAAGAGGGTCAAGAG	2973664	61.16	55
**Multiplex 5**	12	IR-r start (pb)	3551231	**12-r**	TTATACTGGTGCGGAAGCG	3550354	62.38	ATTGGGAACACGAGCTGTG	3551111	62.86	757
	13	IR-l end (pb)	3554066	**13-l**	CTTGGTGGACCTACAGCAC	3554148	62.56	TTCGATGCGTACCAGACTC	3554804	61.71	656
	14	IR-r start (pb)	3710383	**14-r**	ATATCCGTGATCGTCATGGC	3709814	62.21	TGTCCATAACAAGCGGTCG	3710379	62.6	565
		IR-l end (pb)	3711735	**14-l**	CACAAGTACAGCCACACCT	3711772	62.12	GCTCGGGATCGAGTTCATG	3712225	62.81	453
**Multiplex 6**	15	IR-r start (pb)	3795059	**15-r**	TCATTGAGCACGACGATCAC	3794547	62.88	CATTGCATCGGGTGTTCAC	3794902	61.99	355
		IR-l end (pb)	3796411	**15-l**	CTATCGAGCGTTCCACCAC	3796497	62.72	GTCGGTGAATGTGACGTTG	3796752	61.60	255
	16	IR-r start (pb)	3890780	**16-r**	GAAGCTGACCAACCGATAGTC	3890577	62.84	GTGGTGTTCTTGCATGGC	3890732	61.96	155
		IR-l end (pb)	3892132	**16-l**	TTTCGCAGTGATTGCACG	3892321	61.62	CGCATAGCTGTAGCGTCT	3892376	61.87	55
