# Supplementary material for: MLVA Based Classification of Mycobacterium tuberculosis Complex Lineages for a Robust Phylogeographic Snapshot of Its Worldwide Molecular Diversity
Source: PLoS One. 2012 Sep 11;7(9):e41991. doi: 10.1371/journal.pone.0041991 (PMC3439451; doi:10.1371/journal.pone.0041991)
Supplement: Table S2 — (A) Protocol for the MIX preparation of each IS6110AD-typing multiplex. (B) Program cycles used. The process from second to fourth cycle (2*, 3*, 4*) was repeated 35 times. (PDF) [file pone.0041991.s005.pdf]

Supplemental Table S2:
(A) Protocol for the MIX preparation of each IS*6110*AD-typing multiplex. **(B)** Program cycles used for the success of the IS*6110*AD-typing PCR. The process from second to fourth cycle (2*, 3*, 4*) is repeated 35 times.

A

			Concentration (multiplex for 4 amplifications)	Concentration (multiplex for 5 amplifications)	Volume µl (multiplex for 4 amplifications)	Volume µl (multiplex for 5 amplifications)
	Buffer Mix (X)	10	10	10	2.1	2.1
	$MgCl_2$ (mM)	25	25	25	1.68	1.68
Pair of primers 1	Primers F (µM)		16	20	0.394	0.315
	Primers R (µM)		16	20	0.394	0.315
Pair of primers 2	Primers F (µM)		16	20	0.394	0.315
	Primers R (µM)		16	20	0.394	0.315
Pair of primers 3	Primers F (µM)		16	20	0.394	0.315
	Primers R (µM)		16	20	0.394	0.315
Pair of primers 4	Primers F (µM)		16	20	0.394	0.315
	Primers R (µM)		16	20	0.394	0.315
Pair of primers 5	Primers F (µM)			20		0.315
	Primers R (µM)			20		0.315
	DMSO (%)	100	100	100	2.1	2.1
	dNTPs (mM)	25	25	25	0.42	0.42
	rTAQ U/µl	5	5	5	0.084	0.084
	DNA (µg/µl)	0.1	0.1	0.1	1.5	1.5

B

Cycle	Temperature (℃)	Time
1	94	10 min
2*	94	30 sec
3*	58	1 min
4*	72	2 min
5	72	10 min
