# Supplementary material for: MLVA Based Classification of Mycobacterium tuberculosis Complex Lineages for a Robust Phylogeographic Snapshot of Its Worldwide Molecular Diversity
Source: PLoS One. 2012 Sep 11;7(9):e41991. doi: 10.1371/journal.pone.0041991 (PMC3439451; doi:10.1371/journal.pone.0041991)
Supplement: Table S3 — Reclassification of 176 profiles taken from the MIRU-VNTR plus database ( http://www.miru-vntrplus.org/MIRU/index.faces ). (PDF) [file pone.0041991.s006.pdf]

Supplemental Table S3: Re-classification of 176 profiles taken from the MIRU-VNTR*plus* online database (http://www.miru-vntrplus.org/MIRU/index.faces).

ID	Species	Lineage	RD Gagneux's lineage	MIRU12	MIT	Lineage Central Node (MIT)	MIRU12-based Lineage
1805/02	*M. tuberculosis*	Dehli/CAS	East African Indian	224425133533		68	East-African Indian-68
6427/01	*M. tuberculosis*	Dehli/CAS	East African Indian	225426173523		68	East-African Indian-68
7507/01	*M. tuberculosis*	Dehli/CAS	East African Indian	226425193433		68	East-African Indian-68
7747/01	*M. tuberculosis*	Dehli/CAS	East African Indian	225426143523		68	East-African Indian-68
8260/01	*M. tuberculosis*	Dehli/CAS	East African Indian	225424183433		68	East-African Indian-68
9398/01	*M. tuberculosis*	Dehli/CAS	East African Indian	225426173523		68	East-African Indian-68
9915/01	*M. tuberculosis*	Dehli/CAS	East African Indian	225426173523		68	East-African Indian-68
7746/01	*M. tuberculosis*	Dehli/CAS	East African Indian	226225173533	932	93	East Asian-93
2637/02	*M. tuberculosis*	Dehli/CAS	East African Indian	225526173523		101	East Asian-101
7936/01	*M. tuberculosis*	Dehli/CAS	East African Indian	225226173523		101	East Asian-101
3256/02	*M. tuberculosis*	Beijing	East Asian	223325153533	16	16	East Asian-16
3277/02	*M. tuberculosis*	Beijing	East Asian	223325153533	16	16	East Asian-16
3329/02	*M. tuberculosis*	Beijing	East Asian	223325153533	16	16	East Asian-16
3364/02	*M. tuberculosis*	Beijing	East Asian	223325153533	16	16	East Asian-16
4445/02	*M. tuberculosis*	Beijing	East Asian	223325153533	16	16	East Asian-16
4498/02	*M. tuberculosis*	Beijing	East Asian	223325153533	16	16	East Asian-16
4499/02	*M. tuberculosis*	Beijing	East Asian	223325153533	16	16	East Asian-16
3243/02	*M. tuberculosis*	Beijing	East Asian	223325173533	17	17	East Asian-17
4436/02	*M. tuberculosis*	Beijing	East Asian	223325173533	17	17	East Asian-17
3309/02	*M. tuberculosis*	Beijing	East Asian	223325153543	1153	99	East Asian-99
10459/03	*M. tuberculosis*	NEW-1	Euro-American	222325153323	7	7	Euro American-7
12591/02	*M. tuberculosis*	NEW-1	Euro-American	222325163323	111	7	Euro American-7
4192/03	*M. tuberculosis*	Haarlem	Euro-American	222325153323	7	7	Euro American-7
10438/01	*M. tuberculosis*	Cameroon	Euro-American	223215153323	264	12	Euro American-12
10439/01	*M. tuberculosis*	Cameroon	Euro-American	223315153323	12	12	Euro American-12
10445/01	*M. tuberculosis*	Cameroon	Euro-American	223315153323	12	12	Euro American-12
10446/01	*M. tuberculosis*	Cameroon	Euro-American	123215143323		12	Euro American-12
10481/01	*M. tuberculosis*	Cameroon	Euro-American	223315153323	12	12	Euro American-12
1417/02	*M. tuberculosis*	Cameroon	Euro-American	223315153323	12	12	Euro American-12
1428/02	*M. tuberculosis*	Cameroon	Euro-American	223315153323	12	12	Euro American-12
1521/99	*M. tuberculosis*	UgandaII	Euro-American	223315144423		12	Euro American-12
1647/99	*M. tuberculosis*	UgandaII	Euro-American	223315143323	784	12	Euro American-12
2176/99	*M. tuberculosis*	UgandaII	Euro-American	223315143321	1144	12	Euro American-12
2307/99	*M. tuberculosis*	UgandaII	Euro-American	223315144423		12	Euro American-12
5390/02	*M. tuberculosis*	Cameroon	Euro-American	223215153323	264	12	Euro American-12
5429/02	*M. tuberculosis*	Cameroon	Euro-American	223315153323	12	12	Euro American-12
2169/99	*M. tuberculosis*	UgandaI	Euro-American	223225143222		15	Euro American-15
2173/99	*M. tuberculosis*	UgandaI	Euro-American	223225143422		15	Euro American-15
2201/99	*M. tuberculosis*	UgandaI	Euro-American	223325153422		15	Euro American-15
2224/99	*M. tuberculosis*	UgandaI	Euro-American	223325153522	336	15	Euro American-15
2331/99	*M. tuberculosis*	UgandaI	Euro-American	223225153422	1007	15	Euro American-15
2333/99	*M. tuberculosis*	UgandaI	Euro-American	223225153422	1007	15	Euro American-15
3686/03	*M. tuberculosis*	Haarlem	Euro-American	224325153322	32	15	Euro American-15
4217/02	*M. tuberculosis*	Haarlem	Euro-American	224322153322		15	Euro American-15
6946/03	*M. tuberculosis*	Haarlem	Euro-American	224322153322		15	Euro American-15
8750/03	*M. tuberculosis*	Haarlem	Euro-American	226325153322	1094	15	Euro American-15
1850/03	*M. tuberculosis*	LAM	Euro-American	224226143321	738	25	Euro American-25
4412/04	*M. tuberculosis*	X	Euro-American	224325153325	35	33	Euro American-33
5400/02	*M. tuberculosis*	Cameroon	Euro-American	224315153323	27	33	Euro American-33
9787/04	*M. tuberculosis*	X	Euro-American	224325153325	35	33	Euro American-33
10264/03	*M. tuberculosis*	TUR	Euro-American	215125113322	310	40	Euro American-40
10529/03	*M. tuberculosis*	TUR	Euro-American	215125113322	310	40	Euro American-40
11313/03	*M. tuberculosis*	TUR	Euro-American	215125113322	310	40	Euro American-40
1657/03	*M. tuberculosis*	URAL	Euro-American	229225113322		40	Euro American-40
2258/03	*M. tuberculosis*	TUR	Euro-American	215125113322	310	40	Euro American-40
2679/03	*M. tuberculosis*	URAL	Euro-American	239225113322		40	Euro American-40
12637/02	*M. tuberculosis*	Haarlem	Euro-American	225323153323	43	43	Euro American-43
3103/03	*M. tuberculosis*	Haarlem	Euro-American	225323153323	43	43	Euro American-43
2336/02	*M. tuberculosis*	Haarlem	Euro-American	225325153323	45	45	Euro American-45
3342/02	*M. tuberculosis*	Haarlem	Euro-American	225325151323	762	45	Euro American-45
4130/02	*M. tuberculosis*	Haarlem	Euro-American	225325153323	45	45	Euro American-45
4993/02	*M. tuberculosis*	Haarlem	Euro-American	225325153323	45	45	Euro American-45
9532/03	*M. tuberculosis*	Haarlem	Euro-American	225325153323	45	45	Euro American-45
9400/02	*M. tuberculosis*	Haarlem	Euro-American	225324153324		46	Euro American-46
10493/01	*M. tuberculosis*	Ghana	Euro-American	223325143436		86	East Asian-86
2329/99	*M. tuberculosis*	UgandaI	Euro-American	223325133223		101	East Asian-101
8577/03	*M. tuberculosis*	URAL	Euro-American	227225113223	171	101	East Asian-101
2253/99	*M. tuberculosis*	UgandaII	Euro-American	223315143424	821	112	Euro American-112
1571/99	*M. tuberculosis*	UgandaI	Euro-American	223225153423	1264	116	Euro American-116
2111/99	*M. tuberculosis*	UgandaI	Euro-American	223225153423	1264	116	Euro American-116

10469/01	*M. tuberculosis*	Ghana	Euro-American	223325143335		121	Euro American-121
10470/01	*M. tuberculosis*	Ghana	Euro-American	223325143336	673	121	Euro American-121
10486/01	*M. tuberculosis*	Ghana	Euro-American	223325143336	673	121	Euro American-121
10515/01	*M. tuberculosis*	Ghana	Euro-American	223325143336	673	121	Euro American-121
1438/02	*M. tuberculosis*	Ghana	Euro-American	223325143335		121	Euro American-121
2191/99	*M. tuberculosis*	UgandaII	Euro-American	223315143423	11	121	Euro American-121
2197/99	*M. tuberculosis*	UgandaII	Euro-American	223315143423	11	121	Euro American-121
2211/99	*M. tuberculosis*	UgandaII	Euro-American	223315143423	11	121	Euro American-121
2263/99	*M. tuberculosis*	UgandaI	Euro-American	223225143423		121	Euro American-121
2319/99	*M. tuberculosis*	UgandaII	Euro-American	223315143423	11	121	Euro American-121
2379/99	*M. tuberculosis*	UgandaII	Euro-American	223315143423	11	121	Euro American-121
2570/02	*M. tuberculosis*	Ghana	Euro-American	223325143335		121	Euro American-121
2582/02	*M. tuberculosis*	Ghana	Euro-American	223325143335		121	Euro American-121
2597/02	*M. tuberculosis*	Ghana	Euro-American	223325143335		121	Euro American-121
5357/02	*M. tuberculosis*	Ghana	Euro-American	223325143337		121	Euro American-121
8870/03	*M. tuberculosis*	NEW-1	Euro-American	222225143323	547	121	Euro American-121
8885/03	*M. tuberculosis*	LAM	Euro-American	223226141321		128	Euro American-128
946/03	*M. tuberculosis*	LAM	Euro-American	223126153321	536	128	Euro American-128
10581/03	*M. tuberculosis*	LAM	Euro-American	124325153224	326	190	Euro American-190
3262/02	*M. tuberculosis*	LAM	Euro-American	124325153224	326	190	Euro American-190
3310/02	*M. tuberculosis*	LAM	Euro-American	124325143225	325	190	Euro American-190
4431/02	*M. tuberculosis*	LAM	Euro-American	124325143225	325	190	Euro American-190
8078/03	*M. tuberculosis*	LAM	Euro-American	124326153224	140	190	Euro American-190
11046/04	*M. tuberculosis*	S	Euro-American	333325153324	397	212	Euro American-212
1897/04	*M. tuberculosis*	S	Euro-American	333325154324		212	Euro American-212
2151/03	*M. tuberculosis*	S	Euro-American	233325153324	212	212	Euro American-212
2318/06	*M. tuberculosis*	S	Euro-American	332325153325		212	Euro American-212
282/04	*M. tuberculosis*	S	Euro-American	233325133326		212	Euro American-212
3270/04	*M. tuberculosis*	S	Euro-American	233225153326		212	Euro American-212
4526/04	*M. tuberculosis*	S	Euro-American	333225143325	216	212	Euro American-212
6411/05	*M. tuberculosis*	S	Euro-American	333325154325		212	Euro American-212
6424/05	*M. tuberculosis*	S	Euro-American	333325143324	759	212	Euro American-212
742/06	*M. tuberculosis*	S	Euro-American	332325153325		212	Euro American-212
7955/03	*M. tuberculosis*	S	Euro-American	233225153324		212	Euro American-212
8583/04	*M. tuberculosis*	S	Euro-American	333325134222		212	Euro American-212
3995/03	*M. tuberculosis*	LAM	Euro-American	224326153324	215	213	Euro American-213
4428/02	*M. tuberculosis*	LAM	Euro-American	224327153226		213	Euro American-213
7968/03	*M. tuberculosis*	LAM	Euro-American	224326153323	213	213	Euro American-213
9953/04	*M. tuberculosis*	X	Euro-American	224326153322	1062	213	Euro American-213
8915/03	*M. tuberculosis*	EAI	Indo-Oceanic	254326223432	56	56	Indo-Oceanic-56
9267/01	*M. tuberculosis*	EAI	Indo-Oceanic	254326223432	56	56	Indo-Oceanic-56
947/01	*M. tuberculosis*	EAI	Indo-Oceanic	244327221632		56	Indo-Oceanic-56
11359/03	*M. tuberculosis*	EAI	Indo-Oceanic	255316223534		57	Indo-Oceanic-57
6538/03	*M. tuberculosis*	EAI	Indo-Oceanic	254326223533	57	57	Indo-Oceanic-57
7190/03	*M. tuberculosis*	EAI	Indo-Oceanic	254426223533	1003	57	Indo-Oceanic-57
11051/03	*M. tuberculosis*	EAI	Indo-Oceanic	244225223533		59	Indo-Oceanic-59
12778/03	*M. tuberculosis*	EAI	Indo-Oceanic	264225223533	59	59	Indo-Oceanic-59
1797/03	*M. tuberculosis*	EAI	Indo-Oceanic	254326223643		64	Indo-Oceanic-64
6006/03	*M. tuberculosis*	EAI	Indo-Oceanic	244326123413		64	Indo-Oceanic-64
4058/03	*M. tuberculosis*	EAI	Indo-Oceanic	254324223434		69	Indo-Oceanic-69
4850/03	*M. tuberculosis*	EAI	Indo-Oceanic	253327223334		69	Indo-Oceanic-69
1290/03	*M. bovis*	Bovis	M. bovis	232126213322		49	M. bovis
1601/01	*M. bovis*	Bovis	M. bovis	232324253322	49	49	M. bovis
4258/00	*M. bovis*	Bovis	M. bovis	232324253222		49	M. bovis
5346/02	*M. bovis*	Bovis	M. bovis	222324253322	5	49	M. bovis
7072/01	*M. bovis*	Bovis	M. bovis	232324263322		49	M. bovis
751/01	*M. bovis*	Bovis	M. bovis	232324263322		49	M. bovis
7540/01	*M. bovis*	Bovis	M. bovis	232324252322	1321	49	M. bovis
8217/02	*M. bovis*	Bovis	M. bovis	232324243322		49	M. bovis
8490/00	*M. bovis*	Bovis	M. bovis	242424133322		49	M. bovis
951/01	*M. bovis*	Bovis	M. bovis	252324253322		49	M. bovis
9564/00	*M. bovis*	Bovis	M. bovis	232224263222		49	M. bovis
3040/99	*M. canetti*	Canetti	M. canetti	313318672636		60	M.canettii
3041/99	*M. canetti*	Canetti	M. canetti	323212632428	60	60	M.canettii
11443/99	*M. caprae*	Caprae	M. caprae	235424253522		664	West African II
1694/00	*M. caprae*	Caprae	M. caprae	225524253522		664	West African II
1696/00	*M. caprae*	Caprae	M. caprae	136324243522		664	West African II
5358/99	*M. caprae*	Caprae	M. caprae	236224243522		664	West African II
7140/99	*M. caprae*	Caprae	M. caprae	225424253522		664	West African II
7618/99	*M. caprae*	Caprae	M. caprae	246424253622		664	West African II
8319/99	*M. caprae*	Caprae	M. caprae	237324243522		664	West African II
8522/00	*M. caprae*	Caprae	M. caprae	246424323522		664	West African II
8986/99	*M. caprae*	Caprae	M. caprae	246324253422		664	West African II
9062/01	*M. caprae*	Caprae	M. caprae	234323243522	672	664	West African II
9577/99	*M. caprae*	Caprae	M. caprae	236424253522	664	664	West African II
10400/02	*M. africanum*	West African 1	West African-1	224324244221	1282	934	West African I
10458/02	*M. africanum*	West African 1	West African-1	224424243221	348	934	West African I
10473/01	*M. africanum*	West African 1	West African-1	224424243221	348	934	West African I

10480/01	*M. africanum*	West African 1	West African-1	224424243221	348	934	West African I
10494/01	*M. africanum*	West African 1	West African-1	227524242221		934	West African I
1410/02	*M. africanum*	West African 1	West African-1	224424244221	934	934	West African I
5473/02	*M. africanum*	West African 1	West African-1	223424244221		934	West African I
8303/02	*M. africanum*	West African 1	West African-1	224424243221	348	934	West African I
4804/03	*M. africanum*	West African 1	West African-1	224424243221	348	934	West African I
5398/02	*M. africanum*	West African 1	West African-1	224424244221	934	934	West African I
5432/02	*M. africanum*	West African 1	West African-1	224424243221	348	934	West African I
5434/02	*M. africanum*	West African 1	West African-1	223424244221		934	West African I
1443/02	*M. africanum*	West African 1	West African-1	227524242221		934	West African I
1449/02	*M. africanum*	West African 1	West African-1	224424244221	934	934	West African I
1465/02	*M. africanum*	West African 1	West African-1	214324244221		934	West African I
1473/02	*M. africanum*	West African 1	West African-1	223524244221		934	West African I
2569/02	*M. africanum*	West African 1	West African-1	224424243221	348	934	West African I
2577/02	*M. africanum*	West African 1	West African-1	234424244221		934	West African I
3482/03	*M. africanum*	West African 1	West African-1	224424242221		934	West African I
4802/03	*M. africanum*	West African 1	West African-1	224624244221		934	West African I
10462/01	*M. africanum*	West African 2	West African-2	226424243522		664	West African II
10476/01	*M. africanum*	West African 2	West African-2	225424243522	304	664	West African II
10485/01	*M. africanum*	West African 2	West African-2	225424243522	304	664	West African II
10512/01	*M. africanum*	West African 2	West African-2	225424243522	304	664	West African II
10517/01	*M. africanum*	West African 2	West African-2	225324243522		664	West African II
5383/02	*M. africanum*	West African 2	West African-2	225424243522	304	664	West African II
9550/00	*M. africanum*	West African 2	West African-2	237224243421		664-49	West African II-M. bovis
8163/02	*M. africanum*	West African 2	West African-2	227424243522		664	West African II
10514/01	*M. africanum*	West African 2	West African-2	225422244522		934	West African I
5468/02	*M. africanum*	West African 2	West African-2	227424243521		934	West African I
8236/02	*M. africanum*	West African 2	West African-2	224424243622		934	West African I
