# Supplementary material for: MLVA Based Classification of Mycobacterium tuberculosis Complex Lineages for a Robust Phylogeographic Snapshot of Its Worldwide Molecular Diversity
Source: PLoS One. 2012 Sep 11;7(9):e41991. doi: 10.1371/journal.pone.0041991 (PMC3439451; doi:10.1371/journal.pone.0041991)
Supplement: Table S4 — Reclassification of 190 published strains [24] . This collection gathers data from Rio de Janeiro with RDrio deletion (n = 93 strains), international strains with RDrio deletion (n = 7), and other Rio de Janeiro strains which contained the RDrio sequence (n = 90). (PDF) [file pone.0041991.s007.pdf]

Supplemental Table S4: Reclassification of 190 published strains (Lazzarini et al. 2007). This collection gathers data from Rio de Janeiro (n=93 strains), which present the RD^{rio} deletion, some International reference RD^{rio} strains (n=7), and other Rio de Janeiro wild-type strains (n=90).

RD^{rio} strain	12-loci MIRU-VNTR	Newly defined lineages based on 12-loci MIRUs
3	224226153321	Euro American-25
10	224226143321	Euro American-25
14	224226153321	Euro American-25
17	224226133321	Euro American-25
25	224224164321	Euro American-25
32	224226143321	Euro American-25
34	224226143321	Euro American-25
35	225126163311	Euro American-25
37	224216151321	Euro American-25
38	224224133321	Euro American-25
39	224226153321	Euro American-25
40	224226143321	Euro American-25
41	224226143321	Euro American-25
43	224226153321	Euro American-25
44	234225153321	Euro American-25
45	224224133321	Euro American-25
47	224225153321	Euro American-25
48	224225153321	Euro American-25
57	224225163321	Euro American-25
61	224226143321	Euro American-25
67	224226153321	Euro American-25
72	224222163321	Euro American-25
74	224226133321	Euro American-25
75	224226133321	Euro American-25
78	224224133321	Euro American-25
82	224125133321	Euro American-25
84	224226153321	Euro American-25
87	224216151321	Euro American-25
88	224225153321	Euro American-25
91	224225133221	Euro American-25
92	224225143321	Euro American-25
93	224226153321	Euro American-25
42	224115153321	Euro American-34
50	224325143221	Euro American-121
2	223126133321	Euro American-128
6	223226143321	Euro American-128
7	223226152331	Euro American-128
18	222226143321	Euro American-128
22	223226143321	Euro American-128
23	223226153321	Euro American-128
26	223226123321	Euro American-128
27	223224153321	Euro American-128
28	223224153321	Euro American-128
29	223226143321	Euro American-128
29	223226143321	Euro American-128

30	223226163321	Euro American-128
31	223226153321	Euro American-128
33	222226163321	Euro American-128
36	223226163321	Euro American-128
51	223226153321	Euro American-128
54	223124143321	Euro American-128
71	223226153321	Euro American-128
73	223235153321	Euro American-128
77	223226153321	Euro American-128
85	222226153321	Euro American-128
94	222225163321	Euro American-128
1	223125132221	Euro American-163
4	224126152321	Euro American-163
5	223126152311	Euro American-163
8	224126152321	Euro American-163
9	224124132321	Euro American-163
11	222126152321	Euro American-163
13	223126142221	Euro American-163
15	224126142321	Euro American-163
16	224126142321	Euro American-163
19	222226142321	Euro American-163
20	223126142321	Euro American-163
24	225226162321	Euro American-163
46	223126142321	Euro American-163
49	224226152321	Euro American-163
52	224214132321	Euro American-163
53	224225152221	Euro American-163
55	222124142321	Euro American-163
56	223126152321	Euro American-163
58	224126151321	Euro American-163
63	223126152311	Euro American-163
64	222124142321	Euro American-163
65	222124162321	Euro American-163
66	222126152321	Euro American-163
68	224226152321	Euro American-163
76	224224132221	Euro American-163
79	224126152321	Euro American-163
80	224126162321	Euro American-163
81	224126152321	Euro American-163
86	222124142321	Euro American-163
89	222126152321	Euro American-163
90	223126142321	Euro American-163
83	224326163321	Euro American-213
59	225326143221	Euro American-224
60	225326163221	Euro American-224
62	223127151321	Euro American-224
69	124326153321	Euro American-246
70	124326153321	Euro American-246
**International RD^{rio} Strains**		
Percy47 (Djibouti)	224226153321	Euro American-25
14395 (AZ, USA)	224226153321	Euro American-25
15376 (AZ, USA)	224226153321	Euro American-25
2002-1330 (Netherlands)	224226133321	Euro American-25
2002-1384 (Netherlands)	224226133321	Euro American-25
00-817 (IN, USA)	223226153321	Euro American-128

94-1055 (Netherlands)	223124142321	Euro American-163
**Wild type strains**		
144	223115152324	Euro American-8
100	224226153323	Euro American-25
102	224226153323	Euro American-25
109	224226153323	Euro American-25
134	124225133323	Euro American-33
149	224324153124	Euro American-34
163	225213152423	Euro American-42
110	225226153324	Euro American-46
103	223322153323	Euro American-116
112	223226153323	Euro American-128
151	223216143323	Euro American-128
95	124226143224	Euro American-190
96	124326153222	Euro American-190
99	114226153224	Euro American-190
116	123226133225	Euro American-190
117	124326153225	Euro American-190
118	124226153227	Euro American-190
121	124326153222	Euro American-190
122	124326153224	Euro American-190
123	124326152222	Euro American-190
125	125326162227	Euro American-190
126	123226153224	Euro American-190
127	122325133224	Euro American-190
127	122325133224	Euro American-190
128	124326153222	Euro American-190
130	122326153224	Euro American-190
131	123326162224	Euro American-190
132	122326143227	Euro American-190
135	124226162227	Euro American-190
136	123326162227	Euro American-190
137	123326162227	Euro American-190
138	124326143222	Euro American-190
138	124326143222	Euro American-190
139	124226143222	Euro American-190
140	124326143222	Euro American-190
141	124226151223	Euro American-190
142	123327143226	Euro American-190
143	125326153224	Euro American-190
145	123326133224	Euro American-190
146	123326133223	Euro American-190
147	124326152221	Euro American-190
152	124316143222	Euro American-190
153	124226143224	Euro American-190
159	122326153225	Euro American-190
160	124326143222	Euro American-190
161	122326153225	Euro American-190
162	124326153224	Euro American-190
167	124426154222	Euro American-190
167	124426154222	Euro American-190
168	124326153222	Euro American-190
173	123326153222	Euro American-190
174	123326153222	Euro American-190
175	123326153221	Euro American-190

176	123326153222	Euro American-190
177	123326152232	Euro American-190
178	123326143222	Euro American-190
179	224316153228	Euro American-190
180	224316143229	Euro American-190
158	133125161225	Euro American-212
164	333225162322	Euro American-212
165	333225152222	Euro American-212
101	224326153323	Euro American-213
105	224326153323	Euro American-213
106	224326153323	Euro American-213
107	224326153323	Euro American-213
111	224326153323	Euro American-213
113	224326153323	Euro American-213
115	224326153324	Euro American-213
150	224326133324	Euro American-213
157	224316133325	Euro American-213
166	224326153324	Euro American-213
169	224326133322	Euro American-213
170	222326133323	Euro American-213
171	224326133323	Euro American-213
172	223326133323	Euro American-213
181	224326153125	Euro American-213
104	213326153324	Euro American-224
108	223326153324	Euro American-224
148	223316153127	Euro American-224
154	223326153324	Euro American-224
155	223326153324	Euro American-224
156	223326153324	Euro American-224
97	124226153325	Euro American-246
98	124326153324	Euro American-246
114	124326143324	Euro American-246
119	124326133325	Euro American-246
120	124325133326	Euro American-246
124	124326153323	Euro American-246
129	124325133322	Euro American-246
133	124326153325	Euro American-246
