# Supplementary material for: MLVA Based Classification of Mycobacterium tuberculosis Complex Lineages for a Robust Phylogeographic Snapshot of Its Worldwide Molecular Diversity
Source: PLoS One. 2012 Sep 11;7(9):e41991. doi: 10.1371/journal.pone.0041991 (PMC3439451; doi:10.1371/journal.pone.0041991)
Supplement: Table S5 — Results of IS 6110 AD-typing performed on 10 Mozambican strains. Filled square symbolize intact region, empty squares symbolize regions deleted. An asterisk (*) is added when the region size has about 50 bp less than the expected size. (PDF) [file pone.0041991.s008.pdf]

<u>Supplemental Table S5:</u> Results of IS*6110*AD-typing illustrated for selected Mozambican *M. tuberculosis* strains (n=10). Filled square symbolize intact region, empty squares symbolize deleted region. An asterisk (*) is added when the region size has about 50 bp less than the expected size.

**IS*6110* n°**	**1**		**2**		**3**		**4**		**5**		**6**		**7**		**8**		**9**		**10**		**12**	**13**	**14**		**15**		**16**	
Upstream (r) or downstream (l) of the IS*6110*	r	l	r	l	r	l	r	l	r	l	r	l	r	l	r	l	r	l	r	l	r	l	r	l	r	l	r	l
H37Rv	□	■	□	■	■	■	■	■	■	■	■	■	■	□	■	■	■	■	□	■	■	■	■	■	■	■	■	□
1145	□	■	□	■	□	■	■	■	■	■	■	■	■	□	■*	■	■	■	□	□	■	■	■	■	■	■	■	□
1523	□	■	□	■	□	■	■	■	■	■	■	■	■	□	■*	■	■	■	□	□	■	■	■	■	■	■	■	□
1264	□	■	□	■	□	■	■	■	■	■	■	■	■	□	■*	■	■	■	□	□	■	■	■	■	■	■	■	□
1218	□	■	□	■	■	■	■	■	■	■	■	■	■	□	■	■	■	■	□	□	■	■	■	■	■	■	■	□
1369	□	■	□	■	□	■	■	■	■	■	■	■	■	□	■	■	■	■	□	■	■	■	■	■	■	■	■	□
1172	□	■	□	■	□	■	■	■	□	□	■	■	■	□	■	□	■	■	□	■	■	■	□	■	■	■	■	□
1368	■	■	■	■	□	□	■	■	■	■	■	■	■	□	■	■	■	■	□	■	■	■	■	■	■	■	■	□
1371	□	■	□	■	□	□	□	■	■	■	■	■	■	□	■	■	■	■	□	■	■	■	■	■	■	■	■	□
1360	□	■	□	■	□	□	□	■	■	■	■	■	■	□	■	■	■	■	□	■	■	■	■	■	■	■	■	□
1456	□	■	□	■	□	□	□	■	■	■	■	■	■	□	■	■	■	■	□	■	■	■	■	■	■	■	■	□
