# Supplementary material for: MLVA Based Classification of Mycobacterium tuberculosis Complex Lineages for a Robust Phylogeographic Snapshot of Its Worldwide Molecular Diversity
Source: PLoS One. 2012 Sep 11;7(9):e41991. doi: 10.1371/journal.pone.0041991 (PMC3439451; doi:10.1371/journal.pone.0041991)
Supplement: Table S6 — Description of selected regions of difference (RD) located in an adjacent position to an IS 6110 . The first column gives the name of the locus of the two transposases of the concerned IS6110. The second column gives the RD that is adjacent to IS6110 insertion. The third column lists of position of gene(s) involved in the deletion. (PDF) [file pone.0041991.s009.pdf]

<u>Supplemental Table S6</u>: Description of selected regions of difference (RD) located in an adjacent position to an IS*6110*. The first column gives the name of the locus of the two transposases of the concerned IS*6110*. The second column gives the RD that is adjacent to IS*6110* insertion. The third column lists of position of gene(s) involved in the deletion.

**Transposases position into IS*6110***	**Region of difference**	**Position of gene(s) deleted [Reference]**
Rv3186-Rv3187	MiD2	Rv3188-Rv3189 [34]
Rv2354-Rv2355	5	Rv2346c-Rv2353c [35]
Rv2648-Rv2649	11	Rv2645-Rv2659c [35]
Rv1763-Rv1764	14	Rv1765c-Rv1773c [35]
Rv1756c-Rv1757c	152	Rv1754c-Rv1765c [36]
Rv2814c-Rv2815c	207	Rv2814c-Rv2819c and a part of Rv2820c [36]
