# Supplementary material for: MLVA Based Classification of Mycobacterium tuberculosis Complex Lineages for a Robust Phylogeographic Snapshot of Its Worldwide Molecular Diversity
Source: PLoS One. 2012 Sep 11;7(9):e41991. doi: 10.1371/journal.pone.0041991 (PMC3439451; doi:10.1371/journal.pone.0041991)
Supplement: Table S7 — Distribution of phylogenetic groups in the various sub-regions of the world. Percentage of a given group among PGG2/3 isolate is reported in each subregion. (A) Distribution of Haarlem-42/43/45 (B) Distribution of LAM-25/128/163/190/213/224/246 group and these two subgroups LAM-190/213/246 (subgroup A) and LAM-25/128/163/224 (subgroup B). (PDF) [file pone.0041991.s010.pdf]

Supplemental Table S7: Distribution of phylogenetic groups in the various sub-regions of the world. Percentage of a given group among PGG2/3 isolate is reported in each subregion. (A) Distribution of Haarlem-42/43/45. (B) Distribution of LAM-25/128/163/190/213/224/246 group and these two subgroups LAM-190/213/246 (subgroup A) and LAM-25/128/163/224 (subgroup B).

A

Subregions	*Haarlem-42/43/45*
**EURO-S**	26,64
**AMER-S**	23,56
**AFRI-N**	20,9
**EURO-W**	17,33
**EURO-E**	17,16
**EURO-N**	15,32
**ASIA-W**	13,6
**CARI**	12,64
**AMER-N**	11,45
**AFRI-S**	3,28
**AFRI-W**	2,28
**AFRI-E**	1,92
**AFRI-M**	0
**AMER-C**	0

B

Subregions	*LAM-25/128/163/190/213/224/246*	***subgroup A*** *(LAM-190/213/246)*	***subgroup B*** *(LAM-25/128/163/224)*
**AFRI-E**	84,62	13,64	86,36
**AMER-C**	58,33	14,29	85,71
**AMER-S**	43,27	54,81	45,19
**AFRI-S**	39,47	78,27	21,73
**AFRI-N**	37,31	68	32
**CARI**	34,07	10,48	89,52
**EURO-S**	26,25	41,18	58,82
**EURO-W**	23,27	39,72	60,28
**AMER-N**	22,41	54,19	45,81
**EURO-N**	20,56	54,9	45,1
**EURO-E**	7,46	80	20
**ASIA-W**	4	40	60
**AFRI-M**	0	0	0
**AFRI-W**	0	0	0
