# Supplementary material for: MLVA Based Classification of Mycobacterium tuberculosis Complex Lineages for a Robust Phylogeographic Snapshot of Its Worldwide Molecular Diversity
Source: PLoS One. 2012 Sep 11;7(9):e41991. doi: 10.1371/journal.pone.0041991 (PMC3439451; doi:10.1371/journal.pone.0041991)
Supplement: Figure S3 — Result of genotyping on 10 MTC strains (selected from a sample of 100 Mozambican strains). There are 5 distinct genotyping results with each of the primer sets shown; the 1st line shows the classical spoligotyping while the remaining 4 lines show the detection of IS6110 insertional events as detailed in the text. (PDF) [file pone.0041991.s003.pdf]

	Genomic spacer n°	1	2	3	4	5	6	7	8	9	10	11	12	13	14	15	16	17	18	19	20	21	22	23	24	25	26	27	28	29	30	31	32	33	34	35	36	37	38	39	40	41	42	43	44	45	46	47	48	49	50	51	52	53	54	55	56	57	58	59	60	61	62	63	64	65	66	67	68	69	70	71	72	73	74	75	76	77	78	79	80	81	82	83	84	85	86
Strain	Spoligotype43 spacer n°		1	2	3								4	5	6	7			8	9	10	11	12	13	14	15	16	17	18	19	20	21	22	23	24	25	26	27	28	29	30	31	32	33	34		35	36				37	38	39									40	41	42	43																					
H37Rv	Dra*-Drb	■	■	■	■	□	□	□	□	□	□	□	■	■	■	■	□	□	■	■	■	■	■	■	■	□	■	■	■	■	□	□	■	■	■	■	■	■	■	■	■	■	■	□	□	□	□	□	□	□	□	■	■	■	□	□	□	□	□	□	□	□	■	■	■	■	■	□	□	□	□	□	□	□	□	□	□	□	□	■	□	□	□	□	□	■	□
	Dra*-IS3	□	□	□	□	□	□	□	□	□	□	□	□	□	□	□	□	■	□	□	□	□	□	□	□	□	□	□	□	□	□	□	□	■	■	■	■	□	□	□	□	□	□	□	□	□	□	□	□	□	□	■	□	□	□	□	□	□	□	□	□	□	□	□	□	□	□	□	□	□	□	□	□	□	□	□	□	□	□	■	□	□	□	□	□	□	□
	Drb*-IS6	■	□	□	□	□	□	□	□	□	□	□	□	□	□	□	□	□	□	□	□	■	■	□	□	□	□	□	■	□	□	□	□	■	■	□	□	□	□	□	□	□	□	□	□	□	□	□	□	□	□	□	□	□	□	□	□	□	□	□	□	□	□	□	□	□	□	□	□	□	□	□	□	□	□	□	□	□	□	■	□	□	□	□	□	□	□
	Dra*-IS6	□	□	□	□	□	□	□	□	□	□	□	□	□	□	□	□	□	□	□	□	□	□	□	□	□	□	□	□	□	■	■	□	□	■	■	□	□	□	□	□	□	□	□	□	□	□	□	□	□	□	■	■	□	□	□	□	□	□	□	□	□	□	□	■	□	□	□	□	□	□	□	□	□	□	□	□	□	□	■	□	□	□	□	□	□	□
	Drb*-IS3	□	□	□	□	□	□	□	□	□	□	□	□	□	□	□	□	□	□	□	□	□	□	□	□	□	□	□	□	□	□	□	□	□	□	□	□	□	□	□	□	□	■	□	□	□	□	□	□	□	□	□	□	□	□	□	□	□	□	□	□	□	□	□	□	□	□	□	□	□	□	□	□	□	□	□	□	□	□	□	□	□	□	□	□	□	□
1145	Dra*-Drb	■	■	■	■	■	■	□	□	□	□	■	□	□	□	□	□	□	□	□	■	■	■	■	■	■	■	■	■	■	■	■	■	□	□	■	■	■	■	□	□	□	□	■	□	■	■	■	□	■	■	■	■	■	■	■	□	■	■	■	■	■	■	■	■	■	■	■	□	□	□	□	□	□	□	□	□	□	□	■	□	□	□	□	□	□	□
	Dra*-IS3	□	□	□	□	□	□	□	□	□	□	□	□	□	□	□	□	■	□	□	□	□	□	□	□	□	□	□	□	□	□	□	□	□	■	■	■	□	□	□	□	□	□	□	□	□	□	□	□	□	□	□	□	□	□	□	□	□	□	□	□	□	□	□	□	□	□	□	□	□	□	□	□	□	□	□	□	□	□	■	□	□	□	□	□	□	□
	Drb*-IS6	■	□	□	□	□	□	□	□	□	□	□	□	□	□	□	□	□	□	□	□	■	■	□	□	□	□	□	■	□	□	□	■	□	□	□	□	□	□	□	□	□	□	□	□	□	□	□	□	□	□	□	□	□	□	□	□	□	□	□	□	□	□	□	□	□	□	□	□	□	□	□	□	□	□	□	□	□	□	□	□	□	□	□	□	□	□
	Dra*-IS6	□	□	□	□	□	□	□	□	□	□	□	□	□	□	□	□	□	□	□	□	□	□	□	□	□	□	■	■	□	■	■	□	□	■	■	□	□	□	□	□	□	□	□	□	□	□	□	□	□	□	■	■	□	□	□	□	□	□	□	□	□	□	□	□	□	□	□	□	□	□	□	□	□	□	□	□	□	□	□	□	□	□	□	□	□	□
	Drb*-IS3	□	□	□	□	□	□	□	□	□	□	□	□	□	□	□	□	□	□	□	□	□	□	□	□	■	■	□	□	□	□	□	□	□	□	□	□	□	□	□	□	□	□	□	□	□	□	□	□	□	□	□	□	□	□	□	□	□	□	□	□	□	□	□	□	□	□	□	□	□	□	□	□	□	□	□	□	□	□	□	□	□	□	□	□	□	□
1523	Dra*-Drb	■	■	■	■	■	■	□	□	□	□	■	□	□	□	□	□	□	□	□	■	■	■	■	■	■	■	■	■	■	■	■	■	□	□	■	■	■	■	□	□	□	□	■	□	■	■	■	□	■	■	■	■	■	■	■	□	■	■	■	■	□	■	■	■	■	■	■	□	□	□	□	□	□	□	□	□	□	□	■	□	□	□	□	□	□	□
	Dra*-IS3	□	□	□	□	□	□	□	□	□	□	□	□	□	□	□	□	□	□	□	□	□	□	□	□	■	■	□	□	□	□	□	□	□	■	■	■	□	□	□	□	□	□	□	□	□	□	□	□	□	□	□	□	□	□	□	□	□	□	□	□	□	□	□	□	□	□	□	□	□	□	□	□	□	□	□	□	□	□	□	□	□	□	□	□	□	□
	Drb*-IS6	■	□	□	□	□	□	□	□	□	□	□	□	□	□	□	□	□	□	□	□	■	■	□	□	□	□	□	■	□	□	□	■	□	□	□	□	□	□	□	□	□	□	□	□	□	□	□	□	□	□	□	□	□	□	□	□	□	□	□	□	□	□	□	□	□	□	□	□	□	□	□	□	□	□	□	□	□	□	□	□	□	□	□	□	□	□
	Dra*-IS6	□	□	□	□	□	□	□	□	□	□	□	□	□	□	□	□	□	□	□	□	□	□	□	□	□	□	■	■	□	■	■	□	□	■	■	□	□	□	□	□	□	□	□	□	□	□	□	□	□	□	■	□	□	□	□	□	□	□	□	□	□	□	□	□	□	□	□	□	□	□	□	□	□	□	□	□	□	□	□	□	□	□	□	□	□	□
	Drb*-IS3	□	□	□	□	□	□	□	□	□	□	□	□	□	□	□	□	□	□	□	□	□	□	□	□	■	■	□	□	□	□	□	□	□	□	□	□	□	□	□	□	□	□	□	□	□	□	□	□	□	□	□	□	□	□	□	□	□	□	□	□	□	□	□	□	□	□	□	□	□	□	□	□	□	□	□	□	□	□	□	□	□	□	□	□	□	□
1264	Dra*-Drb	■	■	■	■	■	■	□	□	□	□	■	□	□	□	□	□	□	□	□	■	■	■	■	■	■	■	■	■	■	■	■	■	□	□	■	■	■	■	□	□	□	□	■	□	■	■	■	□	■	■	■	■	■	■	■	□	■	■	■	■	■	■	■	■	■	■	□	□	□	□	□	□	□	□	□	□	□	□	■	□	□	□	□	□	□	□
	Dra*-IS3	□	□	□	□	□	□	□	□	□	□	□	□	□	□	□	□	■	□	□	□	□	□	□	□	□	□	□	□	□	□	□	□	□	■	■	■	■	□	□	□	□	□	□	□	□	□	□	□	□	□	□	□	□	□	□	□	□	□	□	□	□	□	□	□	□	□	□	□	□	□	□	□	□	□	□	□	□	□	■	□	□	□	□	□	□	□
	Drb*-IS6	■	□	□	□	□	□	□	□	□	□	□	□	□	□	□	□	□	□	□	□	■	■	□	■	□	■	■	■	□	□	■	■	□	□	□	□	□	□	□	□	□	□	□	□	□	□	□	□	□	□	□	□	□	□	□	□	□	□	□	□	□	□	□	□	□	□	□	□	□	□	□	□	□	□	□	□	□	□	■	□	□	□	□	□	□	□
	Dra*-IS6	□	□	□	□	□	□	□	□	□	□	□	■	□	□	□	□	□	□	□	□	□	□	□	■	□	□	■	□	□	■	■	□	□	■	■	□	□	□	□	□	□	□	□	□	□	□	□	□	□	□	■	□	□	□	□	□	□	□	□	□	□	□	□	□	□	□	□	□	□	□	□	□	□	□	□	□	□	□	■	□	□	□	□	□	□	□
	Drb*-IS3	□	□	□	□	□	□	□	□	□	□	□	□	□	□	□	□	□	□	□	□	□	□	□	■	■	■	□	□	□	□	□	□	□	□	□	□	□	□	□	□	□	□	□	□	□	□	□	□	■	□	□	□	□	□	□	□	□	□	□	□	□	□	□	□	□	□	□	□	□	□	□	□	□	□	□	□	□	□	□	□	□	□	□	□	□	□
1218	Dra*-Drb	■	■	□	□	■	■	■	□	□	□	□	□	□	□	□	□	□	□	□	□	□	□	□	■	■	■	■	■	■	■	■	■	■	■	■	■	■	■	□	□	□	□	■	□	■	■	■	□	■	■	■	■	■	■	■	■	■	■	■	■	■	■	■	■	■	■	□	□	□	□	□	□	□	□	□	□	□	□	■	□	□	□	■	□	□	□
	Dra*-IS3	□	□	□	■	□	□	□	□	□	□	□	□	□	□	□	□	■	□	□	□	□	□	□	□	□	□	□	□	□	□	□	□	□	■	■	■	□	□	□	□	□	□	□	□	□	□	□	□	□	□	□	□	□	□	□	□	□	□	□	□	□	□	□	□	□	□	□	□	□	□	□	□	□	□	□	□	□	□	■	□	□	□	□	□	□	□
	Drb*-IS6	■	□	□	□	□	□	□	□	□	□	□	□	□	□	□	□	□	□	□	□	□	■	□	■	□	□	□	■	□	□	□	□	■	■	□	□	□	□	□	□	□	□	□	□	□	□	□	□	□	□	□	□	□	□	□	□	□	□	□	□	□	□	□	□	□	□	□	□	□	□	□	□	□	□	□	□	□	□	□	□	□	□	□	□	□	□
	Dra*-IS6	□	□	□	■	□	□	□	□	□	□	□	■	□	□	□	□	□	□	□	□	■	□	□	■	□	□	□	□	□	■	■	□	□	■	■	■	□	□	□	□	□	□	□	□	□	□	□	□	□	□	■	□	□	□	□	□	□	□	□	□	□	□	□	■	□	□	□	□	□	□	□	□	□	□	□	□	□	□	□	□	□	□	□	□	□	□
	Drb*-IS3	□	□	□	□	□	□	□	□	□	□	□	□	□	□	□	□	□	□	□	□	□	□	□	□	□	■	□	□	□	□	□	□	□	□	□	□	□	□	□	□	□	□	□	□	□	□	□	□	■	□	□	□	□	□	□	□	□	□	□	□	□	□	□	□	□	□	□	□	□	□	□	□	□	□	□	□	□	□	■	□	□	□	□	□	□	□
1369	Dra*-Drb	■	■	■	□	□	□	□	□	□	□	□	■	■	■	■	□	□	■	■	■	■	■	■	■	■	■	■	■	■	■	■	□	□	■	■	■	■	■	■	■	■	■	□	□	□	□	□	□	□	□	■	■	■	□	□	□	□	□	□	□	□	■	■	■	■	■	□	□	□	□	□	□	□	□	□	□	□	□	□	□	□	□	□	□	■	□
	Dra*-IS3	□	□	□	□	□	□	□	□	□	□	□	□	□	□	□	□	■	□	□	□	□	□	□	□	□	□	□	□	□	□	□	□	□	■	■	■	□	□	□	□	□	□	□	□	□	□	□	□	□	□	□	□	□	□	□	□	□	□	□	□	□	□	□	□	□	□	□	□	□	□	□	□	□	□	□	□	□	□	□	□	□	□	□	□	□	□
	Drb*-IS6	■	□	□	□	□	□	□	□	□	□	□	□	□	□	□	□	□	□	□	□	■	■	□	□	□	□	□	■	□	■	■	□	□	□	□	□	□	□	□	□	□	□	□	□	□	□	□	□	□	□	□	□	□	□	□	□	□	□	□	□	□	□	□	□	□	□	□	□	□	□	□	□	□	□	□	□	□	□	□	□	□	□	□	□	□	□
	Dra*-IS6	□	□	□	□	□	□	□	□	□	□	□	□	□	□	□	□	□	□	□	□	□	□	□	□	□	□	□	□	□	■	■	□	□	■	■	□	□	□	□	□	□	□	□	□	□	□	□	□	□	□	■	□	□	□	□	□	□	□	□	□	□	□	□	□	□	□	□	□	□	□	□	□	□	□	□	□	□	□	□	□	□	□	□	□	□	□
	Drb*-IS3	□	□	□	□	□	□	□	□	□	□	□	□	□	□	□	□	□	□	□	□	□	□	□	□	□	□	□	□	□	□	□	□	□	□	□	□	□	□	□	□	□	□	□	□	□	□	□	□	□	□	□	□	□	□	□	□	□	□	□	□	□	□	□	□	□	□	□	□	□	□	□	□	□	□	□	□	□	□	□	□	□	□	□	□	□	□
1172	Dra*-Drb	■	■	■	□	□	□	□	□	□	□	□	■	■	■	■	□	■	■	■	■	■	■	■	■	■	■	■	■	■	■	■	□	□	■	■	■	■	■	■	■	■	■	□	□	□	□	□	□	□	□	■	■	■	□	□	□	□	□	□	□	□	■	■	■	■	■	□	□	□	□	□	□	□	□	□	□	□	□	■	□	□	□	□	□	■	□
	Dra*-IS3	□	□	□	■	□	□	□	□	□	□	□	□	□	□	□	□	■	□	□	□	□	□	□	□	□	□	□	□	□	□	□	□	□	■	■	■	□	□	□	□	□	□	□	□	□	□	□	□	□	□	□	□	□	□	□	□	□	□	□	□	□	□	□	□	□	□	□	□	□	□	□	□	□	□	□	□	□	□	□	□	□	□	□	□	□	□
	Drb*-IS6	■	□	□	□	□	□	□	□	□	□	□	□	□	□	□	□	□	□	□	□	■	■	□	□	□	□	□	■	□	■	■	□	□	□	□	□	□	□	□	□	□	□	□	□	□	□	□	□	□	□	□	□	□	□	□	□	□	□	□	□	□	□	□	□	□	□	□	□	□	□	□	□	□	□	□	□	□	□	□	□	□	□	□	□	□	□
	Dra*-IS6	□	□	□	■	□	□	□	□	□	□	□	■	□	□	□	□	□	□	□	□	□	□	□	□	□	□	□	□	□	■	■	□	□	■	■	□	□	□	□	□	□	□	□	□	□	□	□	□	□	□	■	■	□	□	□	□	□	□	□	□	□	□	□	□	□	□	□	□	□	□	□	□	□	□	□	□	□	□	□	□	□	□	□	□	□	□
	Drb*-IS3	□	□	□	□	□	□	□	□	□	□	□	□	□	□	□	□	□	□	□	□	□	□	□	□	□	■	□	□	□	□	□	□	□	□	□	□	□	□	□	□	□	□	□	□	□	□	□	□	□	□	□	□	□	□	□	□	□	□	□	□	□	□	□	□	□	□	□	□	□	□	□	□	□	□	□	□	□	□	□	□	□	□	□	□	□	□
1368	Dra*-Drb	■	■	■	■	□	□	□	□	□	□	□	■	■	■	■	□	□	■	■	■	■	■	■	■	■	■	■	■	■	■	■	□	□	■	■	□	□	□	□	□	■	■	□	□	□	□	□	□	□	□	■	■	■	□	□	□	□	□	□	□	□	□	■	■	■	■	□	□	□	□	□	□	□	□	□	□	□	□	□	□	□	□	□	□	□	□
	Dra*-IS3	□	□	□	□	□	□	□	□	□	□	□	□	□	□	□	□	□	□	□	□	□	□	□	□	□	□	□	□	□	□	□	□	□	■	■	□	□	□	□	□	■	□	□	□	□	□	□	□	□	□	□	□	□	□	□	□	□	□	□	□	□	□	□	□	□	□	□	□	□	□	□	□	□	□	□	□	□	□	□	□	□	□	□	□	□	□
	Drb*-IS6	■	□	□	□	□	□	□	□	□	□	□	□	□	□	□	□	□	□	□	□	■	■	□	□	□	□	□	■	□	■	■	□	□	□	□	□	□	□	□	□	□	□	□	□	□	□	□	□	□	□	□	□	□	□	□	□	□	□	□	□	□	□	□	□	□	□	□	□	□	□	□	□	□	□	□	□	□	□	□	□	□	□	□	□	□	□
	Dra*-IS6	□	□	□	□	□	□	□	□	□	□	□	□	□	□	□	□	□	□	□	□	□	□	□	□	□	□	□	□	□	■	■	□	□	■	■	□	□	□	□	□	□	□	□	□	□	□	□	□	□	□	■	□	□	□	□	□	□	□	□	□	□	□	□	□	□	□	□	□	□	□	□	□	□	□	□	□	□	□	□	□	□	□	□	□	□	□
	Drb*-IS3	□	□	□	□	□	□	□	□	□	□	□	□	□	□	□	□	□	□	□	□	□	□	□	□	□	□	□	□	□	□	□	□	□	□	□	□	□	□	□	□	□	□	□	□	□	□	□	□	□	□	□	□	□	□	□	□	□	□	□	□	□	□	□	□	□	□	□	□	□	□	□	□	□	□	□	□	□	□	□	□	□	□	□	□	□	□
1371	Dra*-Drb	■	■	■	■	□	□	□	□	□	□	□	■	■	■	■	□	□	■	■	□	□	□	□	□	□	□	□	□	□	□	□	□	□	□	□	□	□	□	□	□	■	■	□	□	□	□	□	□	□	□	■	■	■	□	□	□	□	□	□	□	□	■	■	■	■	■	□	□	□	□	□	□	□	□	□	□	□	□	□	□	□	□	□	□	□	□
	Dra*-IS3	□	□	□	□	□	□	□	□	□	□	□	□	□	□	□	□	□	□	□	□	□	□	□	□	□	□	□	□	□	□	□	□	□	□	□	□	□	□	□	□	□	□	□	□	□	□	□	□	□	□	□	□	□	□	□	□	□	□	□	□	□	□	□	□	□	□	□	□	□	□	□	□	□	□	□	□	□	□	□	□	□	□	□	□	□	□
	Drb*-IS6	■	□	□	□	□	□	□	□	□	□	□	□	□	□	□	□	□	□	□	□	□	□	□	□	□	□	□	□	□	□	□	□	□	□	□	□	□	□	□	□	□	□	□	□	□	□	□	□	□	□	□	□	□	□	□	□	□	□	□	□	□	□	□	□	□	□	□	□	□	□	□	□	□	□	□	□	□	□	□	□	□	□	□	□	□	□
	Dra*-IS6	□	□	□	□	□	□	□	□	□	□	□	□	□	□	□	□	□	□	□	□	□	□	□	□	□	□	□	□	□	■	■	□	□	□	□	□	□	□	□	□	□	□	□	□	□	□	□	□	□	□	■	□	□	□	□	□	□	□	□	□	□	□	□	□	□	□	□	□	□	□	□	□	□	□	□	□	□	□	□	□	□	□	□	□	□	□
	Drb*-IS3	□	□	□	□	□	□	□	□	□	□	□	□	□	□	□	□	□	□	□	□	□	□	□	□	□	□	□	□	□	□	□	□	□	□	□	□	□	□	□	□	□	□	□	□	□	□	□	□	□	□	□	□	□	□	□	□	□	□	□	□	□	□	□	□	□	□	□	□	□	□	□	□	□	□	□	□	□	□	□	□	□	□	□	□	□	□
1360	Dra*-Drb	■	■	■	■	□	□	□	□	□	□	□	■	■	■	■	□	□	■	■	■	□	■	■	■	■	■	■	■	■	■	■	□	□	□	■	■	□	□	□	□	■	■	□	□	□	□	□	□	□	□	■	■	■	□	□	□	□	□	□	□	□	□	■	■	■	■	□	□	□	□	□	□	□	□	□	□	□	□	□	□	□	□	□	□	□	□
	Dra*-IS3	□	□	□	□	□	□	□	□	□	□	□	□	□	□	□	□	□	□	□	□	□	□	□	□	□	□	□	□	□	□	□	□	□	■	■	■	□	□	□	□	■	□	□	□	□	□	□	□	□	□	□	□	□	□	□	□	□	□	□	□	□	□	□	□	□	□	□	□	□	□	□	□	□	□	□	□	□	□	□	□	□	□	□	□	□	□
	Drb*-IS6	■	□	□	□	□	□	□	□	□	□	□	□	□	□	□	□	□	□	□	□	□	■	□	□	□	□	□	■	■	■	■	□	□	□	□	□	□	□	□	□	□	□	□	□	□	□	□	□	□	□	□	□	□	□	□	□	□	□	□	□	□	□	□	□	□	□	□	□	□	□	□	□	□	□	□	□	□	□	□	□	□	□	□	□	□	□
	Dra*-IS6	□	□	□	□	□	□	□	□	□	□	□	□	□	□	□	□	□	□	□	□	■	■	□	□	□	□	□	□	□	■	■	□	□	□	□	□	□	□	□	□	□	□	□	□	□	□	□	□	□	□	■	□	□	□	□	□	□	□	□	□	□	□	□	□	□	□	□	□	□	□	□	□	□	□	□	□	□	□	□	□	□	□	□	□	□	□
	Drb*-IS3	□	□	□	□	□	□	□	□	□	□	□	□	□	□	□	□	□	■	■	■	□	□	□	□	□	□	□	□	□	□	□	□	□	□	□	□	□	□	□	□	□	□	□	□	□	□	□	□	□	□	□	□	□	□	□	□	□	□	□	□	□	□	□	□	□	□	□	□	□	□	□	□	□	□	□	□	□	□	□	□	□	□	□	□	□	□
1456	Dra*-Drb	■	■	■	■	□	□	□	□	□	□	□	■	■	■	■	□	□	■	■	■	■	■	■	■	■	■	■	■	■	■	■	□	□	□	■	■	□	□	□	□	■	■	□	□	□	□	□	□	□	□	■	■	■	□	□	□	□	□	□	□	□	□	■	■	■	■	□	□	□	□	□	□	□	□	□	□	□	□	□	□	□	□	□	□	□	□
	Dra*-IS3	□	□	□	□	□	□	□	□	□	□	□	□	□	□	□	□	□	□	□	□	□	□	□	□	□	□	□	□	□	□	□	□	□	■	■	■	□	□	□	□	■	□	□	□	□	□	□	□	□	□	□	□	□	□	□	□	□	□	□	□	□	□	□	□	□	□	□	□	□	□	□	□	□	□	□	□	□	□	□	□	□	□	□	□	□	□
	Drb*-IS6	■	□	□	□	□	□	□	□	□	□	□	□	□	□	□	□	□	□	□	□	■	■	□	□	□	□	■	■	□	■	■	□	□	□	□	□	□	□	□	□	□	□	□	□	□	□	□	□	□	□	□	□	□	□	□	□	□	□	□	□	□	□	□	□	□	□	□	□	□	□	□	□	□	□	□	□	□	□	□	□	□	□	□	□	□	□
	Dra*-IS6	□	□	□	□	□	□	□	□	□	□	□	□	□	□	□	□	□	□	□	□	□	□	□	□	□	□	■	□	□	■	■	□	□	□	■	□	□	□	□	□	□	□	□	□	□	□	□	□	□	□	■	□	□	□	□	□	□	□	□	□	□	□	□	□	□	□	□	□	□	□	□	□	□	□	□	□	□	□	□	□	□	□	□	□	□	□
	Drb*-IS3	□	□	□	□	□	□	□	□	□	□	□	□	□	□	□	□	□	□	□	□	□	□	□	□	□	■	□	□	□	□	□	□	□	□	□	□	□	□	□	□	□	□	□	□	□	□	□	□	□	□	□	□	□	□	□	□	□	□	□	□	□	□	□	□	□	□	□	□	□	□	□	□	□	□	□	□	□	□	□	□	□	□	□	□	□	□
